# Neurofilament accumulations in amyotrophic lateral sclerosis patients’ motor neurons impair axonal initial segment integrity

**DOI:** 10.1007/s00018-023-04797-6

**Published:** 2023-05-15

**Authors:** Cynthia Lefebvre-Omar, Elise Liu, Carine Dalle, Boris Lamotte d’Incamps, Stéphanie Bigou, Clément Daube, Léa Karpf, Marc Davenne, Noémie Robil, Coline Jost Mousseau, Stéphane Blanchard, Guillaume Tournaire, Charles Nicaise, François Salachas, Lucette Lacomblez, Danielle Seilhean, Christian S. Lobsiger, Stéphanie Millecamps, Séverine Boillée, Delphine Bohl

**Affiliations:** 1 Sorbonne Université, Institut du Cerveau-Paris Brain Institute-ICM, Inserm, CNRS, AP-HP, Hôpital de la Pitié-Salpêtrière, Paris, France; 2 Université Paris-Cité, CNRS, Saints-Pères Paris Institute for the Neurosciences, Paris, France; 3 GenoSplice Technology, Paris, France; 4 Institut Pasteur, INSERM U1115, Unité Biothérapies pour les Maladies Neurodégénératives, Paris, France; 5 URPhyM, NARILIS, Université de Namur, Namur, Belgium; 6 Département de Neurologie, Assistance Publique Hôpitaux de Paris (APHP), Centre de Référence SLA Ile de France, Hôpital de la Pitié-Salpêtrière, Paris, France; 7 Département de Neuropathologie, AP-HP, Hôpital de la Pitié-Salpêtrière, Paris, France

**Keywords:** Amyotrophic lateral sclerosis, Human induced pluripotent stem cells, Motoneurons, Neurofilaments, Axonal initial segment, Degeneration

## Abstract

Amyotrophic lateral sclerosis (ALS) is the most common motor neuron (MN) disease in adults with no curative treatment. Neurofilament (NF) level in patient’ fluids have recently emerged as the prime biomarker of ALS disease progression, while NF accumulation in MNs of patients is the oldest and one of the best pathological hallmarks. However, the way NF accumulations could lead to MN degeneration remains unknown. To assess NF accumulations and study the impact on MNs, we compared MNs derived from induced pluripotent stem cells (iPSC) of patients carrying mutations in *C9orf72*, *SOD1* and *TARDBP* genes, the three main ALS genetic causes. We show that in all mutant MNs, light NF (NF-L) chains rapidly accumulate in MN soma, while the phosphorylated heavy/medium NF (pNF-M/H) chains pile up in axonal proximal regions of only C9orf72 and SOD1 MNs. Excitability abnormalities were also only observed in these latter MNs. We demonstrate that the integrity of the MN axonal initial segment (AIS), the region of action potential initiation and responsible for maintaining axonal integrity, is impaired in the presence of pNF-M/H accumulations in C9orf72 and SOD1 MNs. We establish a strong correlation between these pNF-M/H accumulations, an AIS distal shift, increased axonal calibers and modified repartition of sodium channels. The results expand our understanding of how NF accumulation could dysregulate components of the axonal cytoskeleton and disrupt MN homeostasis. With recent cumulative evidence that AIS alterations are implicated in different brain diseases, preserving AIS integrity could have important therapeutic implications for ALS.

**Supplementary Information:**

The online version contains supplementary material available at 10.1007/s00018-023-04797-6.

## Background

Amyotrophic lateral sclerosis (ALS) is the most common motor neuron (MN) disease of the adult, leading to paralysis and death within 2–5 years after diagnosis. The most common genetic causes of ALS are hexanucleotide expansions in the *C9orf72* gene responsible for 30–46% of familial ALS (fALS), mutations in the superoxide dismutase 1 (*SOD1*) gene accounting for 12–20% of all fALS and mutations in the *TARDBP* gene (encoding for TDP-43) responsible for ~ 5% of fALS cases [1]. Despite many clinical trials, there is no efficient treatment able to significantly slow down ALS progression, despite some recent promising results from the SOD1 antisense oligonucleotide trial [2]. Therapy development for ALS remains challenging, as reliable diagnostic and prognostic biomarkers are lacking [3]. Recently, neurofilaments (NFs) have emerged as the most promising biomarker for ALS [4, 5] with ongoing studies assessing the promising performances of NFs for both disease diagnostic and prognosis, for improving clinical trial designs and for evaluation of treatment responses [3, 4, 6, 7]. NFs, and especially the light chain (NF-L) and the phosphorylated heavy chain (pNF-H), were detected both in the cerebrospinal fluid (CSF) and in the peripheral blood of ALS patients [3, 8]. As fundamental and specific structural components of neuronal cytoskeletons and the main constituents of MN axons, NF presence in biofluids reflects axon suffering and MN damage [9].

In ALS patients, NF deposits in degenerating MNs have been described since the 1980s. NF accumulations were first suggested by electron microscopy in proximal axon swellings in spinal cord tissues [10–13] and then confirmed by immunostaining with specific antibodies [14–16]. Abnormal NF cytoplasmic inclusions and accumulations in axonal spheroids in surviving MNs is a common histopathological hallmark in all ALS forms [5]. Evidences that these NF accumulations could be detrimental for MNs in ALS came from studies with transgenic mice overexpressing human NF-H or mouse NF-L proteins [17–19] that developed abnormal NF accumulations and axonal degeneration. The discovery of susceptibility variants in the gene encoding NF-H in some ALS sporadic cases also supported a role of NFs in ALS pathology [19]. Studies in the mouse model expressing mutant SOD1^G93A^ showed that NFs accumulated at the pre-symptomatic disease stage, in soma and proximal axonal regions [12], with pathological signs of impaired axonal transport [20]. Strikingly, this proximal region had previously been pointed at in patient’s post-mortem tissues as a region of privileged NF accumulation [21]. While, at that time, the singular protein structure of this axonal proximal region was not described in detail, it is now identified as a specific neuritic domain named the axonal initial segment (AIS), a unique and crucial compartment involved in axon–dendritic polarity [22–26]. Knowing that this AIS region is also involved in action potential (AP) initiation [27] and that MN excitability alteration is an ALS hallmark [28, 29], we asked whether AIS integrity could be altered in ALS MNs and whether it could be correlated to NF accumulations in axonal proximal regions.

To achieve this, we generated human induced pluripotent stem cells (iPSC) from fibroblasts of patients carrying mutations in the three main genes responsible for ALS—*C9orf72, SOD1* and *TARDBP—*as well as isogenic or age and sex-matched control iPSC clones. Our objective was to compare different ALS forms in the same experimental context to identify common alterations, with a specific focus on NF accumulations and AIS alterations. We therefore differentiated iPSC into spinal MNs and showed that the vast majority of produced MNs expressed markers of skeletal MNs able to innervate limb muscles, those primarily affected in ALS patients. Interestingly, our results showed that mutant C9orf72, SOD1 and TARDBP MNs accumulated NF-L in their soma and had fragmented NF-L-positive neurite networks compared to controls. Importantly, when pNF-M/H accumulated specifically in the AIS region of C9orf72 and SOD1 MNs, we could correlate this with strongly altered structural components of the AIS, leading to molecular and geometric disorganizations of the axonal proximal region. Taken together, our results highlight a novel pathway by which NF accumulations disrupt MN homeostasis.

## Methods

### Neuropathology on ALS patient’s spinal cord sections

ALS patients were enrolled in a brain donation program declared by the Ministry of Research and Universities, as requested by French laws. An explicit consent was signed by the patient himself or by the next of kin, in the name of the patient, in accordance with the French Bioethical Laws. Patients are described in the Supplementary Table 1. Three levels of spinal cord (cervical, thoracic, lumbar) were taken. Immunostainings with anti-pNF-H antibodies (SMI31) were performed after deparaffinization of 5 μm-thick sections by an automatic slide stainer (Benchmark XT Ventana staining system). Slides were pre-treated at 95 °C in CC1 (pH 8) proprietary retrieval buffer (Ventana Medical Systems). Antibodies were targeted with a biotin-free detection system (Ventana Medical Systems ultraView Universal DAB Detection Kit).

### Human cells

To establish a human fibroblast collection to generate iPSC, skin biopsies were proposed for French ALS patients, in collaboration with the French National Referral Center for ALS at the Pitié-Salpétriêre hospital (Paris, France) and the Pasteur Institute (project promotor) (Paris, France). The study was conducted in accordance with the Declaration of Helsinki and was approved by an independent ethics committee (“*Comité de Protection des Personnes Île de France VI*”). Fibroblasts carrying the TARDBP^G348C^ mutation were obtained from the Erasmus Hospital (Brussels, Belgium). The study was conducted in accordance with the Declaration of Helsinki and was approved by an independent ethics committee (“*Comité de Protection des Personnes Île de France II*”, DC 2011-534). Written informed consent was obtained from each patient. For control subjects, skin fibroblast cultures were obtained from the Centre de Ressources Biologiques (CRB) of Lyon (France). A statement of all biological samples was made according to French laws formulated by the Research Ministry.

### IPSC generation and characterization

Fibroblasts of a control subject (69 years old), of the patient carrying the N139D mutation in the *SOD1* gene and of the two patients harboring *C9orf72* hexanucleotide repeat expansions, were reprogrammed into iPSC with the integration-free CytoTune™_-_iPS 2.0 Sendai Reprogramming Kit (Thermo Fisher Scientific). For the patient with the G348C mutation in the *TARDBP* gene, reprogramming of fibroblasts was done with integrative retroviral vectors as previously described [30], except that the reprogramming was done with five vectors encoding OCT4, SOX2, KLF4, C-MYC and NANOG. iPSC of a control subject of 33 years old (Ctrl33) were previously generated and described [31]. The control iPSC, named Ctrl40 and Ctrl60, were obtained from the European Collection of Authenticated Cell cultures (HipSci supplier, UK) and derived from fibroblasts of a 40- to 44-year-old woman and a 60- to 64-year-old man (cell lines names HPSI0914i-zerv_8 and HPSI0114i-zapk_3, respectively). Clones were passaged with 0.5 mM UltraPure™ EDTA, pH 8.0 (Thermo Fisher Scientific) and maintained in Essential 8 Medium (Thermo Fisher Scientific) on Geltrex-coated plates. iPSC were regularly tested for recurrent genomic abnormalities by the ICS-digital™ PSC test (Stem Genomics, Montpellier, France) to control cells before initiation of different differentiation protocols.

For characterization, iPSC cultured as embryoid bodies (EBs) for 10 days were plated on Geltrex and cultured for another 10 days in fibroblast medium (DMEM supplemented with 10% fetal bovine serum) to induce spontaneous differentiation. Pluripotency and differentiation potential of iPSC and their EB derivatives were analyzed by immunostaining and PCR with reverse transcription (RT-PCR), as described previously (Supplementary Fig. 1) [30, 31]. When integrative vectors were used for reprogramming, quantitative RT-PCR was performed as described previously [31] to confirm efficient repression of exogenously introduced genes (Supplementary Fig. 2). Genome integrity was assessed by Illumina Human OmniExpress-24 SNP array (300,000 markers) (Integragen, Evry, France) and analyzed using KaryoStudio and GenomeStudio softwares (Illumina). SNP deviations in each iPSC clone from the reference human genome were compared to the original pool of fibroblasts. These analyses confirmed the identity of each clone compared to its parental fibroblast and that cells had not acquired large-scale copy number variations, although a small number of small-scale indels were found (well below the level that would be detected by G-banding). (Supplementary Fig. 3). iPSC were also regularly tested for recurrent genomic abnormalities by the ICS-digital™ PSC test (Stem Genomics, Montpellier, France). We did not detect genomic abnormalities in iPSC clones with these analyses. Genotyping of *SOD1* and *TARDBP* mutations as well as analysis of hexanucleotide repeat expansions in the *C9orf72* gene was performed on genomic DNA isolated from fibroblasts, iPSC clones and MNs, as previously described (Supplementary Fig. 4) [32].

### Gene correction of iPSC

To correct point mutations in iPSC carrying SOD1^N139D^ (clone SOD1-2) and TARDBP^G348C^ (clone TARDBP-1) mutated genes, the CRISPR/Cas9 technology was used [33]. Briefly, iPSC cultured in STEMFLEX medium were dissociated as single cells with Accutase (ThermoFischer Scientific). One million cells were then transfected (4D Nucleofector system, Core Unit AAF-1002B and X unit AAF-1002; Lonza, Switzerland) with a ribonucleoprotein (RNP) complex and 500 pmol of ssODN (single stranded oligodesoxynucleotide) matrix for homologous recombination (ssODN sequences: GCTAGCAGGATAACAGATGAGTTAAGGGGCCTCAGACTACATCCAAGGGAATGTTTATTGGGCGATCCCAATTACACCACAAGCtAAACGACTTCCAGCGTtTCCTGTCTTTGTACTTTC for SOD1; GCCATGATGGCTGCCGCCCAGGCAGCACTACAGAGCAGT TGGGGTATGATGGGCATGTTAGCCAGCCAGCAGAACCAGTCAgGCCCATCGGGTAAcAAtCAAAACCAAGGCAACATGCAG for TARDBP). The RNP complex was prepared with equimolar concentrations of crRNA (crRNA sequences: AGGAGACGCTGGAAGTCGTT for SOD1; TGGTTTTGGTTATTACCCGA for TARDBP) and tracrRNA-ATT0550 (225 pmol of each RNA), with 120 pmol of Cas9 protein (Integrated DNA Technologies (IDT), Iowa, USA). After transfection, all cells were plated and 2 days later ATT0550-positive cells were sorted by FACS (MoFlo Astrios, Beckman Coulter; CYTO-ICAN platform, ICAN Institute, Paris, France) and plated at low concentrations on petri dishes coated with Laminin521 (Stem Cell Technologies) in STEMFLEX medium supplemented with Clone R (Stem Cell Technologies). Seven to 10 days later, individual iPSC clones were picked and expanded in 96-well plates. Each clone was duplicated for either DNA analysis or freezing. Between 100 and 300 clones were picked up. Genetically engineered clones were screened after DNA extraction by PCR amplification of the targeted genomic region and sequencing (primer for PCR: forward: GTAGTGATTACTTGACAGCCC, Reverse: TAATGTTTATCAGGATACATTTCTACAG for SOD1; forward: CTGGCTTTAGATAAATTAATGCT, Reverse: GCTGAATATACTCCACACTGAACA for TARDBP) (primers for sequences: GTAGTGATTACTTGACAGCCC for SOD1; GCCGAACCTAAGCACAATAG for TARDBP). Corrected clones (SOD1-ISO; TARDBP-ISO) were then amplified and characterized at the molecular level. Potential off-target sites with one, two or three mismatches were screened by Sanger sequencing. No off-target mutations were found in the selected clones.

### Differentiation of iPSC into MNs

**Fig. 1:**
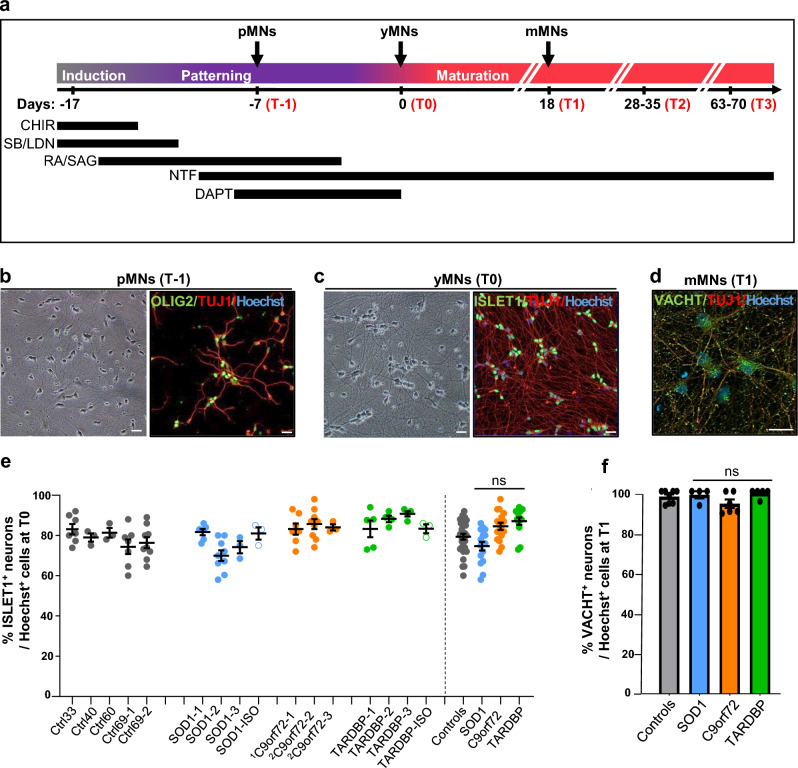
Efficient differentiation of iPSC into spinal MNs. **a** Schematic representation of the differentiation protocol. The different time points of analysis are indicated (*T* − 1, *T*0, *T*1, *T*2 and *T*3). CHIR (CHIR99021), SB (SB431542), LDN (LDN193189), RA (retinoic acid), SAG (smoothened agonist), NTF (neurotrophic factors: BDNF, GDNF, CNTF, IGF1), DAPT (γ secretase inhibitor) are indicated, as well as the different time points of the MN cultures including pMNs (MN progenitors), yMNs (“just born” or young MNs) and mMNs (mature MNs). **b**, **c** Examples of phase contrast microscopy images of pMNs (b, left image) and yMNs (**c**, left image), which show cells with healthy neurite networks at *T* − 1 and *T*0. Right images show representative images of β3-tubulin-positive (TUJ1) neurons co-expressing OLIG2 (**b**) or ISLET1 (**c**). Nuclei are stained with Hoescht H33342. Scale bars: 20 µm. **d** At T1, β3-tubulin-positive (TUJ1) neurons express the vesicular acetylcholine transporter (VACHT) marker. Nuclei are stained with Hoescht H33342. Scale bars: 20 µm. **e**, **f** Quantifications of **e** ISLET1-positive iPSC-derived neurons at *T*0 and **f** VACHT-positive ones at *T*1. Bars: mean ± SEM. *n* = number of independent experiments. **e** Controls (Ctrl33, Ctrl40, Ctrl60, Ctrl69-1, Ctrl69-2): *n* = 29; SOD1 (SOD1-1, SOD1-2, SOD1-3): *n* = 18; SOD1-ISO: *n* = 3; C9orf72 (^1^C9orf72-1, ^2^C9orf72-2, ^2^C9orf72-3): *n* = 19; TARDBP (TARDBP-1, TARDBP-2, TARDBP-3): *n* = 13; TARDBP-ISO: *n* = 3. **f** Controls (Ctrl33, Ctrl69-1, Ctrl69-2, SOD-ISO, TARDBP-ISO): *n* = 8; SOD1 (SOD1-1, SOD1-2): *n* = 4; C9orf72 (^1^C9orf72-1, ^2^C9orf72-2): *n* = 6; TARDBP (TARDBP-1, TARDBP-3): *n* = 5. ns: statistically non-significant by Kruskal–Wallis with Dunn’s test (**e**) and one-way ANOVA with Tukey’s test (**f**)

MNs differentiation was performed as described by Maury et al. [34] with modifications. Briefly, iPSC clones are dissociated with StemPro™ Accutase™ (Thermo Fisher Scientific) and 2.10^5^ cells/ml are suspended into a Neuronal Basic Medium (NBM) supplemented with SB431542 (20 µM, Tocris Bioscience, Bristol, UK), LDN-193189 (0.2 µM, Stemgent) and CHIR99021 (3 µM, Stemgent). NBM is composed of 1:1 DMEM/F-12/Neurobasal™, N2 supplement, B27™ supplement minus vitamin A (all from Thermo Fisher Scientific) and l-ascorbic acid (0.01 μM, Sigma). EBs form within 1 day, and 2 days later the NBM is supplemented with retinoic acid (0.1 µM, Sigma) and SAG smoothened ligand (0.5 µM, Enzo Life Sciences, NY, USA) until day 14. CHIR99021 is removed on day 2, while SB431542 and LDN-193189 are removed on day 4. BDNF and GDNF (10 ng/mL, Miltenyi Biotec) are added from day 7. On day 9, DAPT (10 µM, Sigma) is added and EBs are dissociated the next day with Trypsin–EDTA (Thermo Fisher Scientific) (pMNs, Fig. 1a). Dissociated single cells are then seeded onto coverslips coated either with PEI (polyethyleneimine) solution (2 mg/mL, Sigma) or polyornithine (100 μg/mL, Sigma) and laminin (20 µg/mL, Sigma). Between day 14 and day 17, cells are incubated in NBM with DAPT, BDNF and GNDF. From day 18 (T0, Fig. 1a) and until the end of each experiment, DAPT is removed from NBM, while CNTF and IGF1 are added (10 ng/mL, Miltenyi Biotec) (Fig. 1a). The medium is then changed every other day by replacing only half of the medium to keep a half of the medium conditioned by MNs.

### Immunocytochemistry

Cells were washed in phosphate-buffered saline (PBS) and fixed with 4% paraformaldehyde (PFA) (Euromedex) for 10 min at room temperature (RT). For ion channel staining, cells were fixed for 10 min with PFA 1% (RT) and 10 min in methanol at − 20 °C. Cells were then incubated in a blocking solution (PBS supplemented with 1% bovine serum albumin (BSA), 2% normal goat serum (NGS), 0.1% Triton X-00 for 1 h (RT). Primary antibodies were added, and cells were incubated either for 2 h at RT or overnight at 4 °C. Secondary antibodies were added for 1 h at RT in PBS supplemented with BSA and NGS. Antibodies are listed in Supplementary Tables 2 and 3. Nuclei were stained with Hoechst H33342 (Thermo Fisher Scientific). Slides were mounted with Fluoromount G (Southern Biotech).

### Electrophysiology

Whole-cell patch-clamp recordings were performed on iPSC-derived MNs, derived from SOD1-1, C9orf72-1, TARDBP-1 iPSC clones and control clones. Three days after pMNs dissociation and plating, neurons were incubated (90 min at 37 °C) with lentiviral vectors carrying the cDNA encoding the red fluorescent protein (RFP) under the control of the HB9 promoter [31]. Lentiviral vectors were prepared as previously described [31]. RFP expression was observed a few days later. Only RFP-positive neurons were targeted for patching.

Recordings were performed using an Axopatch 200B amplifier and a Digidata 1440A Analog/Digital interface (Molecular Devices). Data were low-pass filtered at 2 kHz and digitized at a sampling rate of 50 kHz. Patch pipettes were pulled from thin wall borosilicate glass capillaries (Harvard Apparatus) on a Zeitz DMZ puller and had a resistance of 2–3 MΩ when filled with the intracellular solution, containing 10 mM KCl, 130 mM K-gluconate, 2 mM MgCl_2_, 0.1 mM CaCl_2_, 4 mM ATP-Mg, 0.3 mM GTP, 10 mM Na phosphocreatine, 1 mM EGTA and 10 mM HEPES, adjusted to pH 7.2 with KOH (Sigma). The extracellular solution contained 140 mM NaCl, 3 mM KCl, 1 mM MgCl_2_, 2 mM CaCl_2_, 10 mM glucose and 10 mM HEPES adjusted to pH 7.3 with NaOH. Capacitance transients were cancelled out. Series resistance was typically between 5 and 7 MΩ and was compensated by at least 70% for voltage-clamp experiments. Only cells with an Rs ≤ 10 MΩ and a resting membrane potential (RMP) more hyperporlarized than − 35 mV were included in data analysis. Access resistance was monitored at the end of the experiment and cells were discarded if the series resistance changed by more than 20%. In current clamp mode, holding potentials were corrected for a calculated liquid junction potential of 15 mV. RMP was determined by averaging for 30 s of recording and afterward a holding current was used to clamp the resting membrane potential as close as possible to − 70 mV. Whole-cell recordings were performed on control and ALS groups of neurons in parallel on the same day. Electrophysiological recordings were carried out at room temperature (22–24 °C). Data were analyzed off-line using Clampfit 10 software (Molecular Devices) and OriginPro (Origin Lab Corporation). The first AP from the first trace with evoked AP was used to determine the delay, threshold, amplitude and half-width of AP. Firing frequency was obtained by measuring the maximum number of spikes during the current steps protocol (1 s).

### Image analysis

Optical sectioning was obtained using an Imager.M2 equipped with AxioCAM MRm, Apotom.2 module, HXP 120C light source, objectives/NA: 20×/0.8; 40×/1.3; 63×/1.4. Confocal images were obtained with SP8 X WLL, DMI8 stand (Leica). All images were taken at the same laser intensity.

The maximum intensity projection of Z stacks acquired at 63 × was used to measure neuron characteristics with a semi-automatic Fiji Macro. Only cells with a single soma-derived AIS were included in the quantitative analysis. To measure the length or area of the AIS and of the region between the soma and the AIS (the GAP), we used the methods described in Senol et al. [35]. The MAP2 staining allowed us to delimitate the soma contour because MAP2 is a somato-dendritic marker that also stains the beginning of the axon (the GAP) with a decreased intensity (as seen on Fig. 4b, lower panel). Also, both MAP2 and AnkG staining allowed to delimit the GAP, as shown on the scheme (Fig. 4d). On Fig. 5, to plot the soma outline and the GAP, we transiently increased fluorescent signals of both pNF-M/H and panNav stainings. Then, the AnkG-labeled AIS and pNF-M/H accumulations were automatically detected with an intensity threshold defined in control cells. ROIs were registered, source images were reopened and the background was subtracted. The following measures were done automatically: (1) soma, AIS and GAP areas, (2) GAP and AIS lengths, (3) AnkG, pNF-M/H and panNav immunofluorescence integrated density in GAP and AIS regions. The integrated density corresponds to the product of area and mean (immunofluorescence) gray values. GAP and AIS integrated densities were summed to obtain pNF-M/H and panNav distributions from the soma to the end of the AIS. To compare panNav integrated density within the GAP, cells without GAP region were removed. Mean axonal calibers were obtained by dividing GAP and AIS areas by their respective length. We assumed that differences between integrated densities were significant after Kruskal–Wallis with Dunn’s test if the fold difference between control and mutant cells was up to 3. To measure NF accumulation in soma or in neurites, the maximum intensity projection (MIP) of *Z* stacks was used and analysis done with the Fiji software. MN cell bodies were circled by hand on β3-tubulin-labeled images and ROI registered. Second, intensity threshold was set up on NF random images of control MNs. Finally, positive area fraction per cell was calculated. For neurite quantification, we measured on MIP of *Z* stacks the percentage of the total area stained by β3-tubulin, allowing us to compare percentages of β3-tubulin positive area occupied by control neurite networks to those occupied by mutant neurite networks. On each image, MN cell bodies were circled and deleted to measure only the neurite networks.

### RNA extraction and sample preparation for RNA sequencing

Total RNA was extracted using the RNeasy Plus Micro kit (Qiagen) according to the manufacturer’s protocol. RNA concentrations were determined with high-sensitivity RNA ScreenTape on TapeStation (Agilent). cDNA librairies were prepared using KAPA mRNA Hyper Prep kit (Roche) according to the manufacturer’s protocol (150 ng RNA input). Samples were run with an ILLUMINA short-reads sequencer to obtain an average coverage of 30 million of reads per sample (75 bp or 100 bp reads, paired ends). Library preparations and sequencing were performed by the ICM iGeneSeq core facility.

### RNA-seq data analysis

RNA-seq data analyses were performed by GenoSplice technology (www.genosplice.com). Sequencing, data quality, reads repartition (e.g., for potential ribosomal contamination) and insert size estimation were performed using FastQC, Picard-Tools, Samtools and rseqc. Reads were mapped using STARv2.4.0 [36] on the hg19 human genome assembly. Gene expression regulation study was performed as already described [37]. Briefly, for each gene present in the Human FAST DB v2016_1_full annotations, reads aligning on constitutive regions (that are not prone to alternative splicing) were counted. Based on these read counts, normalization and differential gene expression were performed using DESeq2 [38] on R (v.3.2.5). Only genes expressed in at least one of the two compared experimental conditions were further analyzed. Genes were considered as expressed if their fpkm value was greater than 98% the background fpkm value based on intergenic regions. Significant differential expression was defined as adjusted *p* values ≤ 0.05 (P-Adj) after false discovery rate correction and fold changes ≥ 1.5*.* First, we compared controls to each ALS form individually (SOD1, C9orf72 or TARDBP) and calculated differential of gene expressions between yMNs and mMNs. But due to the limited number of patients in the study, we also pooled all samples at each time point to compare all ALS samples (from the 4 patients) to all control samples to identify genes and/or pathways that could be commonly deregulated in the three ALS forms.

### Quantitative PCR

Reverse transcription of 1 µg RNA into cDNA was performed with the SensiFAST cDNA synthesis kit (Bioline, France). Quantitative reverse transcription PCR was then performed in a Roche LightCycler480 using 100 ng of equivalent cDNA and the LightCycler probes master according to Roche procedures. Primers and Taqman probes were from Thermo Fisher Scientific: Hs00544069_m1 and Hs02786624-g1 for CHL1 and GAPDH, respectively.

### Statistical analysis

A minimum of three independent experiments, considered as three differentiation batches from iPSC, was always done. Statistical analysis was performed with GraphPad Prism 8. D’Agostino and Pearson normality test was used to assess Gaussian distribution for large data. For data passing the normality test, one-way ANOVA with Tukey’s multiple comparisons test was performed. For data not passing the normality test, Mann–Whitney test or Kruskal–Wallis with Dunn’s multiple comparisons test were used. When the normality test was not possible, a Gaussian distribution was assumed.

## Results

### Efficient generation of human iPSC-derived MNs from control and ALS individuals

iPSC clones were generated from four patients carrying mutations in *SOD1* (SOD1^N139D^), *C9orf72* (C9orf72, 2 patients) and *TARDBP* (TARDBP^G348C^) genes. Four non-related age- and sex-matched control iPSC clones were included in our analysis (Supplementary Table 4), as well as SOD1 and TARDBP iPSC isogenic controls generated with the CRISPR–Cas9 technology (SOD1-ISO and TARDBP-ISO) (see Methods). iPSC clones were fully characterized at the molecular and functional levels (Supplementary Fig. 1–4). Genome integrity was checked for each iPSC clone (Supplementary Fig. 3).

Each iPSC clone was differentiated into spinal MNs within 17 days (Fig. 1a), as we reported recently [39, 40]. Within 10 days, more than 90% of differentiated iPSC expressed OLIG2, the most specific marker of spinal MN progenitors (pMNs) (Fig. 1b). Seven days later (at T0, Fig. 1a), post-mitotic neurons expressed ISLET1 and HB9 spinal MN markers (Fig. 1c, Supplementary Fig. 5a) and became mature (at T1, Fig. 1a) with the expression of ISLET1, HB9 and TAU and the vesicular acetylcholine transporter (VACHT) 18 days later (mMNs, Fig. 1d. Supplementary Fig. 5a-d). Analysis of the proportions of ISLET1-positive neurons derived from each control and each mutant ALS iPSC clone at T0 (in young or “just born” MNs, yMNs) showed no significant difference between control and ALS clones (Fig. 1e) (all controls (including SOD1-ISO and TARDBP-ISO): 79.1 ± 1.6%; SOD1: 73.5 ± 2.3%; C9orf72: 84.8 ± 1.6%; TARDBP: 83.2 ± 4.3% (mean ± SEM)). At T1, percentages of β3tubulin-positive neurons expressing the VACHT marker were similar between control and ALS iPSC-derived MNs (all controls: 97.4 ± 1.1%; SOD1: 98.3 ± 1.75%; C9orf72: 94.8 ± 2.5%; TARDBP: 98.8 ± 1.4% (mean ± SEM)) (Fig. 1f). Taken together, these data show that ALS MNs were generated with similar kinetics and efficiencies compared to controls.

### iPSC-derived MNs express markers of ALS vulnerable MNs

**Fig. 2:**
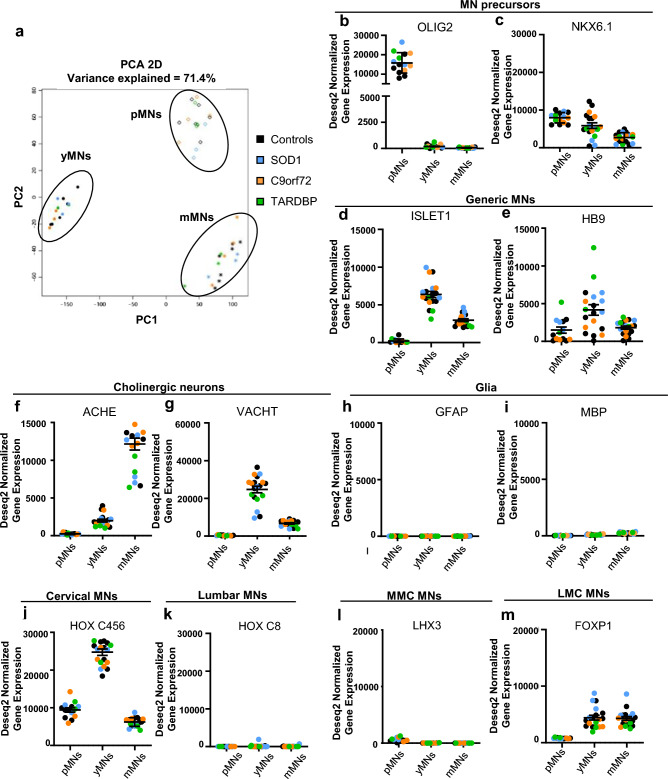
Transcriptome profiling shows that iPSC differentiate into spinal cholinergic neurons expressing markers of MNs of the lateral motor column. RNA sequencing analyses were performed on differentiated cultures at *T* − 1 (pMNs), *T*0 (yMNs) and T1 (mMNs) derived from control (in black), SOD1 (in blue), C9orf72 (in orange) and TARDBP (in green) iPSC clones. **a** Principal component analysis (PCA) shows a time-dependent segregation of yMNs (dots), pMNs (diamonds) and mMNs (stars) samples. **b–m** Graphs show Deseq2 normalized expressions in MNs, at the three time points, of genes encoding **b** *OLIG2*, **c** *NKX6.1* **d** *ISLET1*, **e** *HB9*, **f** acetylcholinesterase (*ACHE*), **g** vesicular acetylcholine transporter (*VACHT*), **h** *GFAP*, **i** *MBP*, **j** *HOX-C4*5*6* markers of the rostral spinal cord MNs, **k** and *HOXC8* a marker of more caudal MNs. **l**, **m** yMNs and mMNs express the transcription factor *FOXP1*, a specific marker of MN of the lateral motor column (LMC), but not the *LHX3* gene encoding a transcription factor involved in specification of MNs located in the median motor column (MMC). For pMN: controls (Ctrl33 *n* = 2; Ctrl69-1 *n* = 2; Ctrl69-2 *n* = 2; SOD1-ISO *n* = 1; TARDBP-ISO *n* = 1), SOD1 (SOD1-1 *n* = 2; SOD1-2 *n* = 1), C9orf72 (^1^C9orf72-1 *n* = 2; ^2^C9orf72-2 *n* = 1), TARDBP (TARDBP-1 *n* = 2). For yMNs: controls (Ctrl33 *n* = 2; Ctrl69-1 *n* = 2; Ctrl69-2 *n* = 2; SOD1-ISO *n* = 1; TARDBP-ISO *n* = 1), SOD1 (SOD1-1 *n* = 2; SOD1-2 *n* = 2), C9orf72 (^1^C9orf72-1 *n* = 2; ^2^C9orf72-2 *n* = 2), TARDBP (TARDBP-1 *n* = 3). For mMNs: controls (Ctrl33 *n* = 2; Ctrl69-1 *n* = 1; Ctrl69-2 *n* = 1; SOD1-ISO *n* = 1; TARDBP-ISO *n* = 1), SOD1 (SOD1-1 *n* = 2; SOD1-2 *n* = 2), C9orf72 (^1^C9orf72-1 *n* = 2; ^2^C9orf72-2 *n* = 2), TARDBP (TARDBP-1 *n* = 3)

In ALS patients, it is well established that the different MN subtypes are not equally affected during disease progression [41, 42]. Thus, it was crucial for us to first define which MN subtypes were generated in our cultures to assure that our further results will be relevant to ALS. Thus, we performed RNA sequencing of pMNs, yMNs and mMNs derived from control iPSC clones (Ctrl33, Ctrl69-1, Ctrl69-2, SOD1-ISO, TARDBP-ISO) and from different clones of the four ALS patients (SOD-1, SOD-2, C9orf72-1.1, C9orf72-2.1, TARDBP-1). Principal component analysis (PCA) showed efficient segregation of cells according to their maturation status (Fig. 2a). Then, the RNA expression patterns of differentiation markers were analyzed. First, expression of the two most specific transcription factors of MN progenitors, *OLIG2* and *NKX6.1* [43, 44], were detected in all control and ALS pMNs (Fig. 2b, c). Whereas *OLIG2* expression was detected only in progenitors [45], *NKX6.1* was detected in progenitors and in post-mitotic MNs as described previously [46]. Other less specific markers of MN progenitors were also expressed in pMNs including *NKX6.2, NGN1, NGN2 and PAX6* (Supplementary Fig. 6a), whereas markers of interneuron progenitors were not or barely detected (*DBX1, DBX2, FOXN4, NGN3*) (Supplementary Fig. 6b) [44]. Second, efficient differentiation of pMNs into post-mitotic MNs was confirmed with expressions of *ISLET1* and *HB9* transcription factors (Fig. 2d, e) [47]. HB9 was also detected in progenitors in agreement with its critical role for the proper specification of MNs during development [48, 49]. Importantly, markers of V1, V2 and V3 spinal interneurons (expressing *Engrailed 1*, *CHX10* and *NKX2.2* transcription factors, respectively) and markers of ISLET1-positive interneurons (expressing TLX3 and BRN3A) [44] were not detected in yMNs and mMNs (Supplementary Fig. 6c). Third, the maturation of MNs into cholinergic neurons was confirmed by the detection of transcripts encoding acetylcholinesterase (*ACHE*) (Fig. 2f) and *VACHT* (Fig. 2g), while we did not detect any expression of genes encoding enzymes involved in the synthesis of other neurotransmitters (Supplementary Fig. 6d), except glutaminase 1 (*GLS1*), the enzyme known to produce glutamate. This result agrees with previous work showing that a single MN can release both acetylcholine and glutamate at the mixed MN and Renshaw cell synapse [50]. The skeletal identity of iPSC-derived cholinergic neurons was also confirmed by showing no or barely detectable expression of markers specific of cholinergic visceral MNs and cholinergic interneurons [44] [51] (Supplementary Fig. 6e). Fourth, glial markers were barely detectable and mostly absent. Analyzed markers included genes expressed in astrocytes (*GFAP, ALDHL1, S100β, EAAT1*), oligodendrocytes (*MBP, SOX10, MOG*) and microglia (*CD11b, IBA1*) (Fig. 2h, i; Supplementary Fig. 6f). Immunofluorescent labeling did not allow detecting GFAP, MBP or Iba1 expression in MN cultures. Fifth, MNs expressed an HOX code typical of the cervical spinal cord with high expression of *HOXC456* and no expression of the lumbar marker *HOXC8* (Fig. 2j, k). Sixth, we observed that *LHX3* expression, a marker of MNs innervating epaxial muscles, was negative in MNs (Fig. 2l), while *FOXP1* expression, a marker of MNs innervating limb muscles (LMC), was detected in all control and ALS MNs (Fig. 2m; Supplementary Fig. 6g). Other markers of LMCs were also detected including *Paxillin* and *NCK2* [52, 53] (Supplementary Fig. 6h). Finally, we looked for the expression of recently identified markers of alpha MNs, which were shown to be more vulnerable in ALS, compared to gamma MNs [54, 55]. Expression of alpha-MN markers, although low, was clearly higher than expression of gamma-MN markers (Supplementary Fig. 6i, j). This alpha identity of MNs was confirmed by expression of different markers of muscle fibers (Supplementary Fig. 6k). In conclusion, this detailed analysis showed that cervical cholinergic skeletal MNs were generated in our cultures with the specific expression of alpha MN markers suggesting that cultures contained MNs able to innervate limb muscles, those being affected in ALS patients, and thus being of particular interest to study ALS MN phenotypes.

### Neurofilament accumulations in soma and proximal neurite regions of ALS MNs

**Fig. 3:**
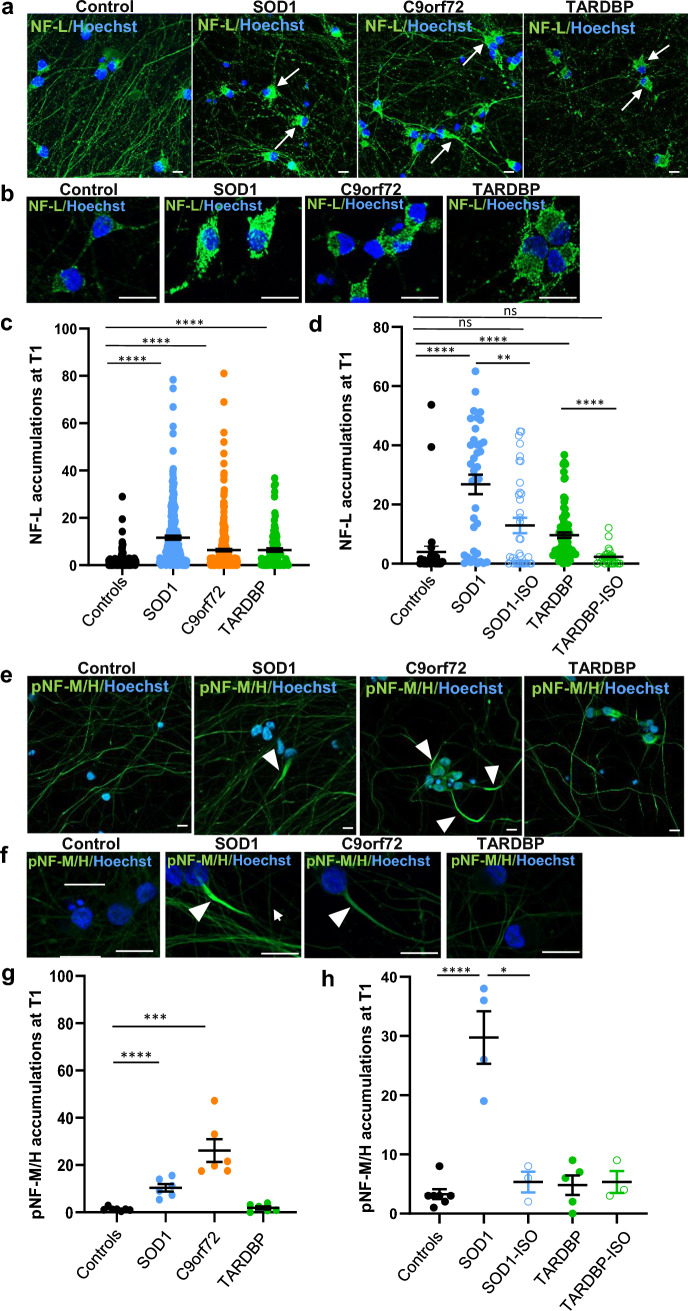
NF-L and pNF-M/H defects in ALS iPSC-derived MNs. **a**, **b**, **e**,** f** Representative images show control, mutant SOD1, C9orf72 and TARDBP iPSC-derived mMNs labeled with antibodies directed against the light neurofilament subunit NF-L (**a**, **b**) or the phosphorylated form of the medium–heavy neurofilament subunit pNF-M/H (**e**, **f**). Images in **a** and **e** show large fields of MN cultures at low magnification, while images in **b** and **f** are at higher magnifications. Scale bars: 10 µm. Nuclei were stained with Hoescht H33342. In **a**, arrows indicate NF-L accumulations in soma. In **e**, **f**, arrowheads indicate pNF-M/H increased staining in neurite proximal regions. **c**, **d** Quantifications of soma NF-L signal intensity in MNs (arbitrary units) derived from control and ALS clones. In **c**, the median intensity per control MN was 0.19. In SOD1, C9orf72 and TARDBP MN cultures, 93.5%, 88% and 79.5% of MNs had a higher median intensity than 0.19, respectively. Controls (Ctrl33, Ctrl69-1, Ctrl69-2): *N* = 399, *n* = 8; SOD1 (SOD1-1, SOD1-2) *N* = 245, *n* = 6; C9orf72 (^1^C9orf72-1, ^2^C9orf72-2) *N* = 375, *n* = 6; TARDBP (TARDBP-1, TARDBP-3): *N* = 169, *n* = 4. In **d**, significant differences of NF-L accumulations were shown between SOD1 MNs and isogenic SOD1-ISO MNs, and between TARDBP MNs and isogenic TARDBP-ISO MNs. Controls (Ctrl69-2): *N* = 33, *n* = 3; SOD1 (SOD1-2): *N* = 37, *n* = 3; SOD1-ISO: *N* = 37; *n* = 3; TARDBP (TARDBP-1, TARDBP-3): *N* = 169, *n* = 4; TARDBP-ISO: *N* = 20, *n* = 3. Statistical significance by Kruskal–Wallis with Dunn’s test (****p < 0.0001, **p < 0.01). *n* = number of independent experiments. *N* = total number of analyzed neurons. **g**, **h** Quantifications of pNF-M/H accumulations at T1 in MNs derived from control and ALS clones showing significative differences between (**g**) control MNs and SOD1 MNs or C9orf72 MNs, and (**h**) SOD1 MNs and isogenic SOD1-ISO MNs. Each dot represents the percentage of neurons with pNF-M/H accumulations counted in one experiment. 200–300 neurons were analyzed per experiment. *n* = number of independent experiments. In **g**: controls (Ctrl33, Ctrl69-1, Ctrl69-2): *n* = 7; SOD1 (SOD1-1, SOD1-2) *n* = 6; C9orf72 (^1^C9orf72-1, ^2^C9orf72-2) *n* = 6; TARDBP (TARDBP-1, TARDBP-3) *n* = 6. In **h**: controls (Ctrl33, Ctrl69-1, Ctrl69-2): *n* = 7; SOD1 (SOD1-1, SOD1-2): *n* = 6; SOD1-ISO: *n* = 3; TARDBP (TARDBP-1, TARDBP-3): *n* = 6; TARDBP-ISO: *n* = 3. Mean ± SEM. Statistical significance by unpaired *t* test (*p < 0.05; ***p < 0.001; ****p < 0.0001)

To assess whether NFs accumulated in ALS MNs, iPSC differentiated cultures were immunolabeled to identify NF-L and pNF-M/H proteins. For NF-L, soma accumulations were already observed in SOD1 and C9orf72 yMNs (Supplementary Fig. 7a) and with time in culture these accumulations increased significantly in all 3 mutant ALS forms compared to controls (Fig. 3a, b) as shown by quantitative analyses (Fig. 3c, d; Supplementary Fig. 7c for individual data). For pNF-M/H, early soma accumulations were observed in SOD1 and C9orf72 yMNs, as for NF-L (Supplementary Fig. 7b), but with time pNF-M/H soma accumulations became rarer while it accumulated in the neurites (Fig. 3e, f). We measured significantly higher numbers of MNs with pNF-M/H accumulations in their neurite proximal regions in mutant SOD1 and C9orf72 MNs (Fig. 3g, h. Supplementary Fig. 7d for individual data). These proximal pNF-M/H accumulations were not observed in TARDBP MNs (Fig. 3e, f). In parallel, the NF-L staining showed that neurite networks of control and ALS MNs were healthy with no neurite fragmentation at T0 (Supplementary Fig. 7a), while at T1 ALS neurite networks were less dense with bead-like structures all along neurites (Fig. 3a, Supplementary Fig. 7e), indicating neurite fragmentation and degeneration. In parallel, the pNF-M/H neurite staining remained homogenous in MNs at T0 and T1 (Fig. 3e, Supplementary Fig. 7b), suggesting that the different NF subunits were differently altered during the degenerative process.

Pathological pNF-M/H signs were reminiscent of those previously described in MNs of post-mortem spinal cord ALS patient tissues [11, 14, 15, 56]. Thus, to assess whether similar accumulations could specifically be observed in neurite proximal regions of spinal MNs of human patients, post-mortem tissues with mutations in the three main ALS genes were stained for pNF-H (Supplementary Table 1). In all three patients, we observed large NF inclusions in soma and numerous neurite NF beadings (Supplementary Fig. 8a–d). Interestingly, some of these NF beadings were localized in neurite proximal regions in agreement with our observations in iPSC-derived human ALS MNs.

### Comparisons of transcriptome profiles between ALS and control MNs

To look for commonly deregulated genes between SOD1, C9orf72, TARDBP and control MNs, we used RNA sequencing (Fig. 2a). In pMNs and yMNs, no gene was found significantly dysregulated between mutant and control MNs (fold change ≥ 1.5 and adjusted P value ≤ 0.05). Then, we compared the differential gene deregulations with time between yMNs and mMNs in each mutant versus control MNs (Supplementary Table 5–8). Despite the identification of interesting deregulated genes involved in protein degradation, cell survival and metabolism, none was shared between ALS forms. Thus, we compared all ALS mMNs to control mMNs (at the mature state) and found seven upregulated genes (Table 1, Supplementary Table 9). The most deregulated gene encoded the cell adhesion molecule L1-like protein (CHL1). Its increased expression levels in ALS MNs vs control MNs were confirmed by quantitative RT-PCR (Supplementary Fig. 9). This gene was of particular interest as CHL1 belongs to the L1 subfamily of the Ig superfamily cell adhesion molecules, which share a highly conserved region containing an ankyrin-binding site. Interestingly, CHL1 was shown to interact with ankyrin G [57], the AIS master organizer protein [22, 58], and to be able to recruit it to the plasma membrane [59]. CHL1 was also shown to be involved in neuronal migration, survival, neurite outgrowth and regeneration after injury [60, 61].

**Table 1:** Genes upregulated in ALS mMNs vs control mMNs (fold change ≥ 1.5; adjusted *p* value ≤ 0.05)

Gene symbol	Gene name	Belongs to significant GO terms	Known functions	Fold change	Adjusted *p* value	References
CHL1	Cell adhesion molecule L1-like protein	Extracellular exosome Proteinaceous extracellular matrix Cell adhesion Plasma membrane	Recruits AnkG to the plasma membrane Cholinergic synapse formation	362.8	2.75 × 10^–6^	[22, 58–61]
CTSF	Cathepsin F	Extracellular space Extracellular exosome	Lysosomal enzyme Role in intracellular degradation and turnover of proteins	357.0	1.17 × 10^–3^	[117]
RIMBP2	RIMS-binding protein 2	Plasma membrane	Role in synaptic transmission	29.5	1.41 × 10^–3^	[118]
NNAT	Neuronatin		Disease development and progression	876.5	3.77 × 10^–3^	[119]
DACH1	Dachshund family transcription factor 1		Transcription factor	4.2	4.36 × 10^–3^	
HSPA1L	Heat shock protein family A (Hsp70) member 1-like	Cell body	In conjunction with other HSP, this protein stabilizes existing proteins against aggregation and mediates the folding of newly translated proteins in the cytosol and in organelles	3.9	6.71 × 10^–3^	[120]
TCF4	Transcription factor 4		Transcription factor	3.7	6.71 × 10^–3^	

### AIS alterations in MNs with pNF-M/H accumulations

**Fig. 4:**
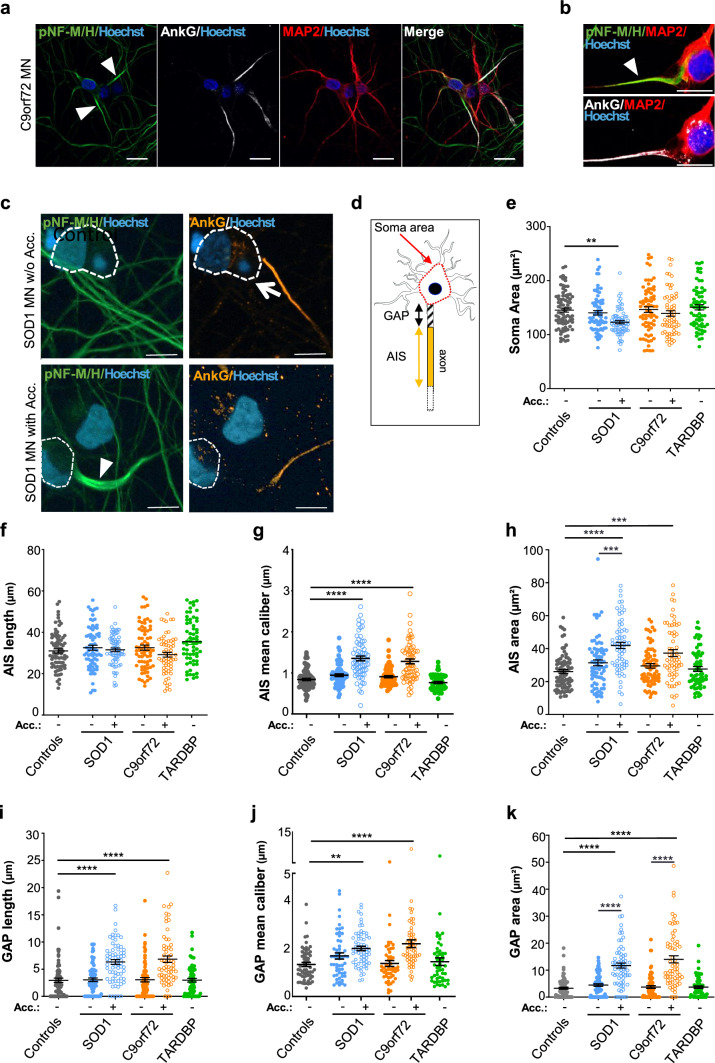
Structural alterations of the proximal axonal regions in mutant SOD1 and C9ORF72 iPSC-derived MNs in the presence of pNF-M/H accumulations. **a**, **b** Representative images of C9orf72 MNs co-immunostained with antibodies against pNF-M/H, AnkG and MAP2, to identify axonal pNF-M/H accumulation, AnkG-positive AIS and MAP2-positive dendrites, respectively. Low levels of MAP2 expression were detected in axons compared to dendrites, in agreement with previous reports showing that MAP2 is enriched in dendrites, but not fully specific as its MAP2c isoform detected by the antibody (Synaptic Systems) can also be present in axons [116]. Scale bars: 20 µm. A confocal image of pNF-M/H accumulation is shown in **b**. Nuclei were stained with Hoescht H33342. Arrowheads indicate pNF-M/H accumulations. Scale bars: **a** 20 µm, **b** 10 µm. **c** Representative images of SOD1 MNs with or without (w/o) pNF-M/H accumulation (Acc.) labeled with antibodies against pNF-M/H and AnkG. Dotted lines indicate soma localizations. Nuclei were stained with Hoescht H33342. Scale bars: 10 µm. **d** Drawing representing an MN and its different regions of interest: the soma area, the GAP and the AIS along the proximal axonal region. The red dotted line indicates how the soma was delimitated in each neuron with concave lines crossing both sides of each neurite entry zone, as described previously [35]. **e**–**k** Graphs show the different quantifications done on images acquired in cultures of control, SOD1, C9orf72 and TARDBP MNs with or without pNF-M/H accumulations (Acc) (see “Materials and methods”): **e** soma areas, **f**–**k** length, mean axonal caliber and area of AIS (**f**, **g**, **h**) and GAP (**i**, **j**, **k**). Statistical significance shown are either between ALS and control MNs or between MNs with or without accumulation by Kruskal–Wallis with Dunn’s test (**p < 0.01, ***p < 0.001, ****p < 0.0001). *n* = number of analyzed MNs from three independent experiments. Controls (Ctrl33, Ctrl69-2): *n* = 81; SOD1 (SOD1-1, SOD1-2): *n* = 72 (with acc.)/*n* = 68 (without acc.); C9orf72 (^1^C9orf72-1, ^2^C9orf72-2): *n* = 63 (with acc.)/*n* = 71 (without acc.). TARDBP (TARDBP-1, TARDBP-2): *n* = 64. See Supplementary Fig. 10 for data in individual clones

To determine if the AIS was altered in ALS MNs and if it could be a target of NF accumulations, we first determined if pNF-M/H accumulated in axonal regions where AIS is localized. Control and ALS MNs were co-immunostained with antibodies against MAP2, a microtubule-associated protein enriched in dendrites and soma, AnkG (ankyrin G) and pNF-M/H, to identify MAP2-negative axons, the AIS and NFs, respectively. Results showed that the pNF-M/H intensity staining was higher in proximal axons where the AIS-specific AnkG staining was also localized (Fig. 4a, b). Interestingly, the abnormal pNF-M/H accumulation was always localized in the region between the soma and the AIS (Fig. 4c), called the axon hillock (AH), and partially also invaded the AIS. As the AH region, essentially made of microtubules, has no known specific marker and thus cannot be specifically identified, and as its integrity seemed altered, we called the region between the soma and the AIS the “GAP” for the quantification analysis (Fig. 4d).

Soma areas, without pNF-M/H accumulations, were similar in size between control and mutant SOD1, C9orf72 and TARDBP MNs (Fig. 4e; Supplementary Fig. 10e). In the presence of pNF-M/H accumulations, we measured significantly smaller soma areas in SOD1 MNs derived from one out of the two analyzed clones (Supplementary Fig. 10e) compared to control MNs. However, the weak difference between both SOD1 clones could suggest that these SOD1 MNs begin suffering, compared to other mutant MNs. Next, we measured the lengths (Fig. 4f, i; Supplementary Fig. 10f, i), mean calibers (Fig. 4g, j; Supplementary Fig. 10g, j) and areas (Fig. 4h, k; Supplementary Fig. 10h, k) of AIS and GAP in the different MN cultures. Interestingly, while AIS lengths were similar between the different MN populations with or without pNF-M/H accumulations (Fig. 4f, Supplementary Fig. 10f), GAP lengths increased significantly in SOD1 and C9orf72 MNs with pNF-M/H accumulations (Fig. 4i, Supplementary Fig. 10i), showing that MNs accumulating pNF-M/H had an AIS displaced towards a distal position when compared to control MNs. Also, calibers of both AIS and GAP were significantly increased when MNs contained pNF-M/H accumulations in their proximal axonal region (Fig. 4g, j, Supplementary Fig. 10g, j).

**Fig. 5:**
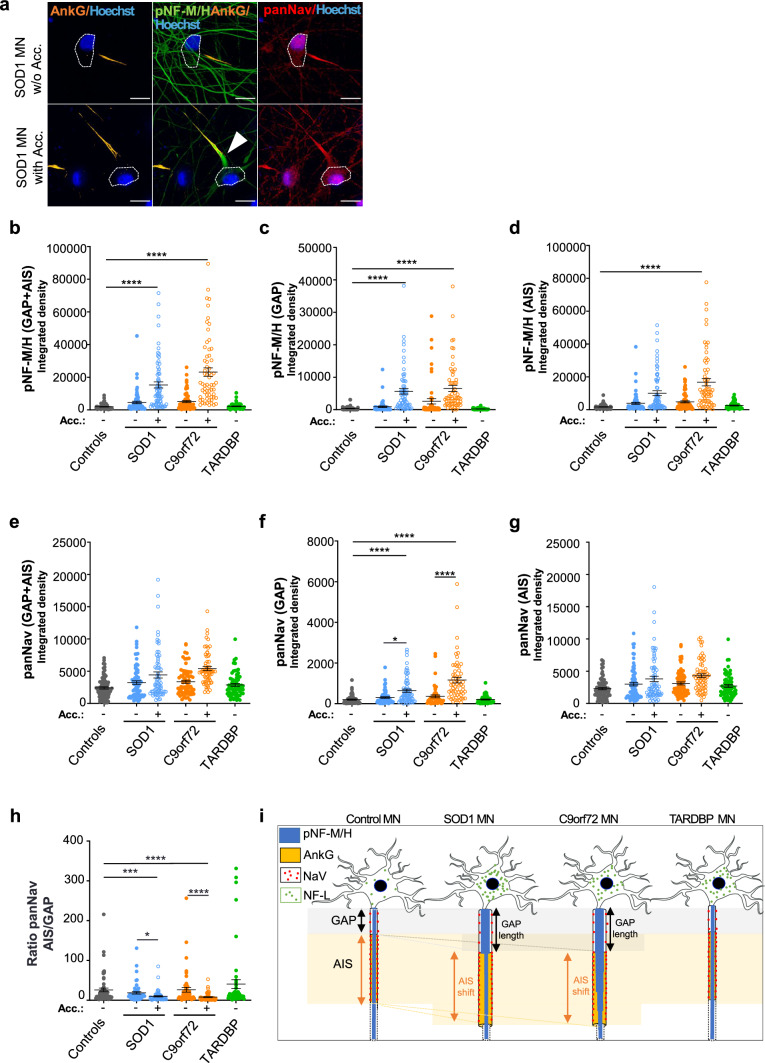
Mislocalization of Nav channels in the GAP of mutant SOD1 and C9orf72 iPSC-derived MNs in the presence of pNF-M/H accumulations. **a** Representative images of SOD1 MNs with or without (w/o) pNF-M/H accumulation (Acc.) labeled with antibodies against pNF-M/H, AnkG and panNav. Dotted lines show soma localization and the arrowhead indicates pNF-M/H accumulation. Nuclei were stained with Hoescht H33342. Scale bars: 10 µm. **b**–**h** Integrated densities of pNF-M/H and panNav signals were calculated to assess their distributions in the GAP and the AIS. **b**–**d** pNF-M/H integrated density measured **b** between the soma and the end of the AIS (GAP + AIS), **c** in the GAP and **d** in the AIS. **e**–**g** panNav integrated density measured **e** between the soma and the end of the AIS (GAP + AIS), **f** in the GAP and **g** in the AIS. **h** Ratio of panNav integrated density in the AIS to the GAP. *n* = number of analyzed MNs from three independent experiments per lines. Controls (Ctrl33, Ctrl69-2): *n* = 81. ALS without accumulation: SOD1 (SOD1-1, SOD1-2): *n* = 74, C9orf72 (C9orf72-1, ^2^C9orf72-2): *n* = 71, TARDBP (TARDBP-1, TARDBP-2) *n* = 71. ALS with accumulation: SOD1 (SOD1-1, SOD1-2): *n* = 69, C9orf72 (C9orf72-1, ^2^C9orf72-2): *n* = 63. Kruskal–Wallis with Dunn’s test (mean ± SEM). Statistical significance shown either between ALS and control MNs or between MNs with or without accumulation. ****p < 0.0001, ***p < 0.001, *p < 0.05. See Supplementary Fig. 11 for data in individual clones. **i** Schematic representation of control and ALS MNs, with pNF-M/H (in blue), the GAP (in white), the AnkG-positive AIS (in orange), Na channels (red dots) and soma NF-L (green dots). In the presence of pNF-M/H accumulations, we measured: increased GAP lengths, a distal shift of the AIS, increased axon areas and calibers in SOD1 and C9orf72 axons compared to TARDBP and control axons, and Nav channels increase in the GAP. In TARDBP MNs, neither pNF-M/H accumulation nor AIS alterations were observed, but they showed NF-L soma accumulation and neurite decrease, as in SOD1 and C9orf72 MNs (drawing based on images from Servier Medical Art (http://smart.servier.com/))

Next, we analyzed pNF-M/H and voltage-gated sodium (Nav) channel repartitions along the axonal proximal region of MNs. Indeed, Nav channels could be affected by pNF-M/H accumulations, as the AIS is highly enriched in these channels that are anchored to AnkG [62, 63]. Immunofluorescent integrated densities of pNF-M/H and panNav were measured in the GAP and along the entire AIS using a triple immunostaining with antibodies directed against AnkG, pNF-M/H and panNav (Fig. 5a). In the entire GAP + AIS region, the pNF-M/H integrated density was significantly increased in both SOD1 and C9orf72 MNs compared to control MNs (Fig. 5b; Supplementary Fig. 11b). Whereas in C9orf72 MNs accumulations were seen in both GAP and AIS regions, in SOD1 MNs the pNF-M/H accumulations were observed either only in the GAP or in the GAP and the AIS, a phenotype that is clone dependent (Fig. 5d; Supplementary Fig. 11d). Quantifications of panNav integrated densities showed that Nav channels were mostly localized in the AIS region (Fig. 5f, g; Supplementary Fig. 11f, g) and that the increased panNav integrated densities seen in one SOD1 clone and the two C9orf72 clones, in the presence of pNF-M/H accumulations (Fig. 5e; Supplementary Fig. 11e) were due to the increase of Nav channels in the GAP rather than in the AIS (Fig. 5f, g; Supplementary Fig. 11f, g). Thus, increased panNav integrated densities in the GAP (Fig. 5f; Supplementary Fig. 11f) and significantly lower ratios of panNav in the AIS vs in the GAP in the presence of pNF-M/H accumulations (Fig. 5h; Supplementary Fig. 11 h) suggest a modified distribution of Nav channels in pathological conditions.

Overall, our data show that the molecular organization and the geometry of the axonal proximal region were strongly modified in the presence of pNF-M/H accumulations, with a distal delocalization of the AIS, a GAP increase of Nav channels, as well as increased axonal calibers and axonal areas of the entire region GAP/AIS region in iPSCs-derived SOD1 and C9orf72 MNs (Fig. 5i). In TARDBP MNs, no alteration of the AIS or of Nav channel localization was observed in this region. Taken together, our results provide, for the first time, strong evidence of a correlation between the presence of pathological axonal pNF-M/H accumulations in ALS MNs and alterations of the AH and AIS regions that are crucial for MN polarity, transport and excitability.

### ALS MNs become hyperexcitable with time

**Fig. 6:**
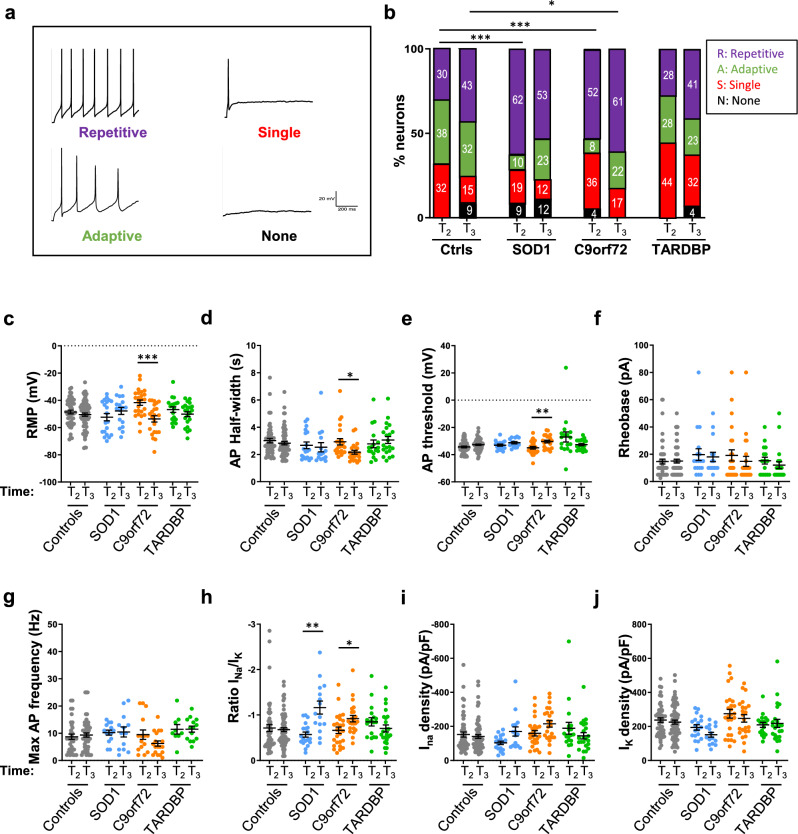
Electrophysiological properties are altered in ALS motor neurons. Whole-cell patch-clamp recordings of iPSC-derived MNs from control subjects and ALS patients at T2 and T3. Recordings were done on the largest RFP-positive neurons visualized after gene transfer with a lentiviral vector encoding RFP under the control of the HB9 MN-specific promoter. *n* = number of recorded MNs from two or three independent experiments. Controls (Ctrl69-1, Ctrl69-2): T2, *n* = 51; T3, *n* = 74; SOD1-2: T2, *n* = 18; T3, *n* = 15; ^1^C9orf72-1: T2, *n* = 24; T3, *n* = 22; TARDBP-1: T2, *n* = 17; T3, *n* = 21. **a**–**g** Current-clamp recordings. **a** AP firing patterns of iPSC-derived MNs in response to injected currents (step 1 s, 5–100 pA) ranged in four groups: none, one spike, adaptive firing and repetitive firing. **b** Percentages of ALS and control MNs per firing pattern at T2 and T3. White numbers represent percentages of firing MNs. Statistical significances are shown for the adaptive AP firing groups between control and SOD1 MNs at T2, and between control and C9orf72 MNs at T2 and T3 (Fisher’s exact test with adjusted *p* value. *p < 0.5; ***p < 0.001). **c** Resting membrane potential (RMP), **d** AP half-width, **e** AP threshold, **f** rheobase, **g** maximum frequency. **h**–**j** Voltage-clamp recordings. **h** Ratio of Na^+(peak)^ and K^+(low)^ current densities during voltage steps of various amplitudes. **i** Na^+(peak)^ current density. **j** K.^+(low)^ current density. **C**–**j** Kruskal–Wallis with Dunn’s test (*p < 0.05, **p < 0.01, ***p < 0.001) shows significant differences between T2 and T3

As the AIS is directly involved in AP initiation [22] and as altered excitability is a common phenomenon reported in ALS patients [28, 29], we next asked whether the electrophysiological properties of ALS iPSC-derived MNs were altered with time in culture. While spontaneous APs were rarely recorded, most of the control and ALS MNs were able to fire at least one AP in response to injected currents at late time points (T2 and T3, Fig. 1a) (Fig. 6a, b). When we compared AP distributions with respect to four different firing patterns (no AP, single AP, adaptive AP, repetitive AP firing) [64] (Fig. 6a), no significant differences in the distributions of AP were measured at T2 and T3 between all ALS and control MNs (Supplementary Fig. 12a). However, when mutant MN cultures were analyzed separately, the proportions of MNs with adaptive AP were significantly lower than those in control MNs in mutant SOD1 and C9orf72 MNs at T2 and at both T2 and T3, respectively (Fig. 6b). These decreased proportions were concomitant with increased proportions of mutant MNs with repetitive AP firing compared to control MNs. Then, AP parameters were measured, including RMP (resting membrane potential), AP half-width, AP threshold, rheobase, max AP frequency, the delay of first AP, AP peak amplitude and AP amplitude. We measured significant differences in RMP, AP half-width and AP threshold measures, but only in C9orf72 MNs compared to SOD1, TARDBP and control MNs between T2 and T3, while the other parameters remained unchanged (Fig. 6c–g; Supplementary Fig. 12b-d). Next, we performed voltage-clamp recordings. In this mode, the recorded inward current had the fast activation and inactivation characteristics of voltage-gated sodium (Na) channel, while the outward current had slow activation and no inactivation of the voltage-gated potassium (K) channel. Interestingly, recordings showed that *I*_Na_/*I*_K_ density ratio significantly increased with time in SOD1 and C9orf72 MNs, while the ratio had a trend to decrease in TARDBP MNs (Fig. 6h). Analysis of Na and K current densities showed no significant alterations of Na and K current densities in ALS MNs (Fig. 6i, j). Taken together, these data suggest that mutant C9orf72 and SOD1 MNs acquire some excitability defects with time in culture, a phenotype in agreement with AIS alterations.

## Discussion

In the present study, we compared phenotypic defects between mutant SOD1, C9orf72, TARDBP and control MNs and identified specific alterations in ALS MNs. The ALS MNs were generated with the same efficiencies and kinetics as control MNs and characterized as MNs with characteristics of MN innervating limb muscles. With time in culture, ALS MNs showed signs of degeneration, with NF-L accumulations in their soma, as well as fragmented neurites. Interestingly, when pNF-M/H accumulated in proximal axonal regions of C9orf72 and SOD1 mMNs, major alterations in the axonal proximal region were measured altering the axon hillock (or the GAP) and AIS molecular and geometric organizations. Neither pNF-M/H accumulations nor AIS modifications were observed in TARDBP MNs. Moreover, at late time points, SOD1 and C9orf72 MNs progressively acquired excitability alterations. Taken together, these results suggest a strong correlation between pNF-M/H accumulations and AIS alterations. These results expand our understanding of how NF accumulations could dysregulate components of the axonal cytoskeleton, and in particular of the AIS, an axonal sub-compartment crucial for polarity and excitability of MNs.

The RNA sequencing analysis we performed on iPSC-derived MNs allowed to characterize MN subtypes present in our cultures, to confirm that they were representative models of ALS vulnerable MNs. We showed that progenitors expressed markers of the pMN domain of the embryonic spinal cord [43] and that post-mitotic MNs were skeletal, cholinergic and able to fire AP. Moreover, the protocol allowed the generation of MN subtypes with an HOX cervical identity and expressing LMC- and alpha-MN markers, specific of limb-innervating MNs, the MNs that are primarily affected in ALS patients [47, 65]. Our analysis also showed that control and ALS MNs shared similar expression patterns, suggesting that the different defects observed between SOD1, C9orf72 and TARDBP MNs were not related to different MN subtypes present in the cultures. However, it cannot be completely excluded that phenotypic differences may be related to unknown intrinsic differences between MN cultures. For example, as MNs were generated without muscle cells to focus our study on MN intrinsic defects, MNs were probably not fully mature and thus could be in different intermediate states that we cannot identify.

To our knowledge, our study is the first one showing ALS phenotypic defects in iPSC-derived MNs characterized by their specific expression of LMC and alpha MN markers. This is an important step forward in the field of ALS modeling with human iPSC, as generation and analysis of both affected and not affected MNs in the same culture could mask disease-specific phenotypic differences. The mechanisms underlying the different MN death susceptibilities in ALS remain not fully understood, but it is highly likely that the vulnerability of certain MNs to degeneration is related to anatomical specificities and their transcriptome profiles [41, 66]. Therefore, studies analyzing specific MN subtypes affected in the disease can strengthen and provide clearer interpretations of results.

A hallmark of many neurodegenerative diseases is the presence of protein inclusions in post-mortem tissues of patients. Despite years of research there is still a debate on whether aggregates are protective or deleterious to MNs. In ALS iPSC-derived MN models, abnormal protein accumulations were rarely described [67, 68] and were always observed in young MNs, suggesting that they could be involved early in the pathological process. In the present study, we analyzed by immunofluorescence whether SOD1, C9orf72 and TARDBP MNs accumulated abnormal proteins. We were able to observe accumulations of misfolded SOD1 proteins in soma of SOD1 MNs compared to control MNs (Supplementary Fig. 5e). This observation is consistent with Shi et al. who showed that the N139D mutation can destabilize rapidly the SOD1 protein conformation leading to an accelerated rate of aggregation [69]. In TARDBP MNs, we detected very rare nuclear TDP-43 dots and soma inclusions of the phosphorylated form of TDP-43 (Supplementary Fig. 5f, g). Interestingly, TDP-43 and SOD1 proteins were shown to interact and modulate NF-L mRNA stability [70], and recently DPRs were suggested to promote TDP-43 aggregation [71]. Taken together, these studies suggest that in SOD1, C9orf72 and TARDBP MNs, the expression of NF-L subunits could be directly or indirectly altered by mutant proteins, and this could explain the abnormal NF-L accumulations in soma and decreased NF-L expression in neurites, we observed in ALS MNs. Besides the impact of mutated proteins on NF-L, mutant SOD1 and DPRs could also have a direct impact on the AIS stability through their interactions with specific Nav subunits known to interact with AnkG [72, 73].

NFs abnormalities have been so far only described in few studies of iPSC-derived MNs from two SOD1 patients [74], two TARDBP patients [75] or one TARDBP and two sporadic cases [76]. None of these studies analyzed AIS alterations. The strength of the present study is that we made the choice to look for NF defects in the three main ALS forms (with mutations in SOD1, C9orf72 and TARDBP genes), with the idea that comparing three different mutated genes in a same experimental context could bring more information on differences between ALS forms. Our study showed that NF-L was accumulating in soma and was reduced in neurites of SOD1, C9orf72 and TARDBP MNs compared to control MNs. To our knowledge, this is the first report showing such detailed NF-L alterations in ALS MNs. As NF-L was shown to play a role in axonal transport [77, 78], our results suggest that these alterations could be part of transport defects in ALS MNs. Recently, NF-L was shown to be involved in the regulation of organelle trafficking in iPSC-derived MNs [79], and this could be due to its interaction with the myosin-Va motor protein [80]. Evidence of such transport defects were originally suggested from observations in ALS patients [12, 77, 81, 82], and more recently from studies that provided evidence for axonal transport deficits in ALS MNs in animal and iPSC models [20, 67, 68, 83–90]. With NF-L being crucial for correct NF heteropolymer formation and axonal cytoskeletal stability [78], NF-L neuritic alterations could be early signs of neurodegeneration and would lead to NF-L leakage. This would require an assay to measure NF-L in culture supernatants to assess if it is possible to mimic with iPSC-derived MNs the in vivo situation of increased NF-L levels in blood of ALS patients [3, 8].

We also show that pNF-M/H accumulated in axonal proximal region of SOD1 and C9orf72 MNs. As it is well known that NF-M and NF-H phosphorylation is crucial for NF stability [91], other consequences of altered NF-L expression could be an impact on NF heteropolymer assembly (which requires a strict stoichiometry of each NF subunit) and the abnormal accumulations of phosphorylated NF-M and NF-H. Thus, a disorganization of the cytoskeleton and of its interactions with other axonal components including microtubules [92] could have also an impact on the stability of the AIS which is anchored onto microtubules. Consistent with this hypothesis, it is known that the different constituents of the cytoskeleton, including NFs and microtubules, interact with cytolinker proteins that connect the different elements of the cytoskeleton with each other [93]. Among them, the plectin protein was identified in the MNs of the spinal cord [94] and shown to bind to microtubules and each NF subunit [95]. According to BIOGRID (the Biological General Repository for Interaction Datasets), plectin could also interact with AnkG. Taken together, these data suggest that pNF-M/H could interact with plectin, AnkG and the microtubules, thereby creating a complex in which the alteration of one component could have an impact on the others.

Moreover, we show that pNF-M/H accumulation is strongly correlated with several alterations in molecular and geometric organizations of both AH and AIS, suggesting that all the axonal trafficking, before and after the AIS, could be disturbed. Indeed, the AIS plays an essential role in the axo-dendritic polarity regulating protein mobility and vesicular trafficking between the soma and the axon, with both a surface and cytoplasmic diffusion barrier [25, 58, 96]. In parallel, we did not observe any alteration of AIS organization in axonal proximal regions in mutant TARDBP MNs, a result that strengthens a possible direct link between the presence of pNF-M/H accumulations in this region and AIS alterations. However, we cannot exclude that absence of this AIS phenotype in TARDBP MNs could be due to the specific mutation we studied or to a time lag of phenotype onset. This latter hypothesis is in line with Fujimori et al., suggesting a time-dependent initiation of different cell death mechanisms in FUS and TARDBP iPSC-derived MNs [97] and consistent with our results. Indeed, we showed different timings of phenotype appearances with pNF-M/H invading both AH and AIS, followed by electrophysiological defects within C9orf72 MNs, while SOD1 MNs had milder electrophysiological defects with pNF-M/H invading only the AH.

The AIS is a highly structured axonal segment that has crucial roles in neuron homeostasis. An essential role of the AIS in neurons is that it is the unique axonal region where APs are generated and shaped before their propagation along the axon [27, 98]. MNs, like other neuronal types, display a specific AIS plasticity and geometry, responsible for their excitability properties, which depend on AIS length, its distance from the soma and its combination and distribution of voltage-gated ion channel isoforms [99–102]. Interestingly, we showed such AIS alterations in ALS MNs, with the AIS shifting distally, combined with increased axonal calibers and modified distribution of Nav channels in SOD1 and C9orf72 MNs. Moreover, our electrophysiological recordings suggested excitability modifications in more mature SOD1 and C9orf72 MNs, resulting agreement with the findings assessing a correlation between an AIS distal shift and neuron excitability [101, 102]. Taken together, these data strongly suggest that AIS alterations observed in mutant SOD1 and C9orf72 MNs could explain excitability defects. However, with iPSC models, MNs mature progressively and in our experiments AIS alterations were observed at a time point when MNs were not able to fire AP, suggesting that AIS alterations could occur independently of any electric activity. In agreement with this hypothesis, individual NF-M and NF-H phosphorylation was shown to play a role as signals for their assembly and organization within neurons [93]. Thus, increased phosphorylation of NF-M and NF-H could be a response to an NF-L altered expression, and also to the abnormal accumulation of proteins in the AH because of axonal transport defects. The increased Nav channels we measured in the GAP could be part of this accumulation and be linked to their increased endocytosis [22]. However, our experiments do not allow us to conclude whether excitability alterations are secondary or primary events in the pathology, which remains an ongoing debate due to conflicting results in the literature [103]. Nevertheless, our data agree with recent reports on MN diseases, showing AIS plastic changes around symptom onset in the SOD1^G127X^ mouse model [104, 105], and altered AP waveform and shorter AIS in mutant VRK1 iPSC-derived MNs of patients with hereditary motor neuropathy associated with upper MN signs [106]. However, none of these reports established, as we do in the present study, a correlation with one of the most established human ALS pathological signs, NF accumulations.

At the molecular level, we were interested to look for common deregulated genes between ALS forms and controls. When we analyzed each ALS form separately and compared differences between yMNs and mMNs, only few deregulated genes were identified and none was in common. However, when comparing all different ALS mMNs as a pool to control mMNs, we identified upregulation of the CHL1 gene. Interestingly, CHL1 encodes a protein able to bind and to recruit AnkG to the plasma membrane [57, 59]. A hypothesis would be that the CHL1 upregulation, measured in SOD1 and C9orf72 MNs, would occur in response to AIS geometry and plasticity alterations due to progressive accumulation of pNF-M/H and subsequent obstruction of the AH. Moreover, it was shown that increasing CHL1 expression could promote ALS MN neuritogenesis and survival in response to degeneration [60, 61], which could help explain why CHL1 increases in TARDBP MNs that showed no AIS alterations. Strengthening the early involvement of AIS alterations in ALS pathology, two transcriptomic studies revealed that the AnkG gene transcription was downregulated in whole spinal cord grey matter post-mortem tissues of five patients with sporadic ALS and two patients with familial ALS, including one SOD1 patient with an A4V mutation [107] and in human motor nerves (obtained from diagnostic biopsies) of eight sporadic ALS patients, at a time representing an early phase of the disease [108].

Interestingly, during the past decade, several studies have assessed if AIS plasticity and alterations of its components could be implicated in neurodegenerative and psychiatric diseases, as well as in brain trauma [22, 58, 109–111]. For example, in a mouse model of Alzheimer’s disease (AD), amyloid-β plaques were shown to disrupt AIS with reduction of their numbers and lengths [112]. Also, Sohn et al*.* showed that the frontotemporal dementia (FTD)-causing V337M mutation in MAPT impaired structural AIS plasticity, leading to excitability modifications in iPSC-derived neurons from FTD patients [113]. Here, we go one step further and show a strong correlation between AIS alterations, pNF-M/H accumulations and ALS pathology, and we suggest that one target for ALS therapy could include preservation of the AIS structure. Consistent with this idea, Schafer et al. showed that calpain inhibitors can preserve the AIS after ischemic injury [114], and recently Zhang et al. showed that mDia1, a formin, can contribute to maintain AIS composition and structure [115]. Whether these approaches would work for ALS remains to be determined.

### Supplementary Information

Below is the link to the electronic supplementary material.

Supplementary file1 (PDF 3223 KB)

Supplementary file2 (XLSX 92 KB)

Supplementary file3 (XLSX 65 KB)

Supplementary file4 (DOCX 46 KB)

## Data Availability

The datasets used and/or analyzed during the current study are available from the corresponding author on reasonable request. The raw RNA-seq data were uploaded to the NCBI GEO. Information to have access to these data are provided in the file “link to supporting data”.

## Abbreviations

AD: Alzheimer disease
AIS: Axonal initial segment
ALS: Amyotrophic lateral sclerosis
AnkG: Ankyrin G
CSF: Cerebrospinal fluid
FTD: Fronto-temporal dementia
iPSC: Induced pluripotent stem cells
MN: Motor neuron
NF: Neurofilaments
SOD1: Superoxide dismutase 1
TARDBP: TAR DNA-binding protein
