# Supplementary material for: Neurofilament accumulations in amyotrophic lateral sclerosis patients’ motor neurons impair axonal initial segment integrity
Source: Cell Mol Life Sci. 2023 May 15;80(6):150. doi: 10.1007/s00018-023-04797-6 (PMC10185656; doi:10.1007/s00018-023-04797-6)
Supplement: Supplementary file 1 — Supplementary file1 (PDF 3223 KB) [file 18_2023_4797_MOESM1_ESM.pdf]

Supplementary Fig.1

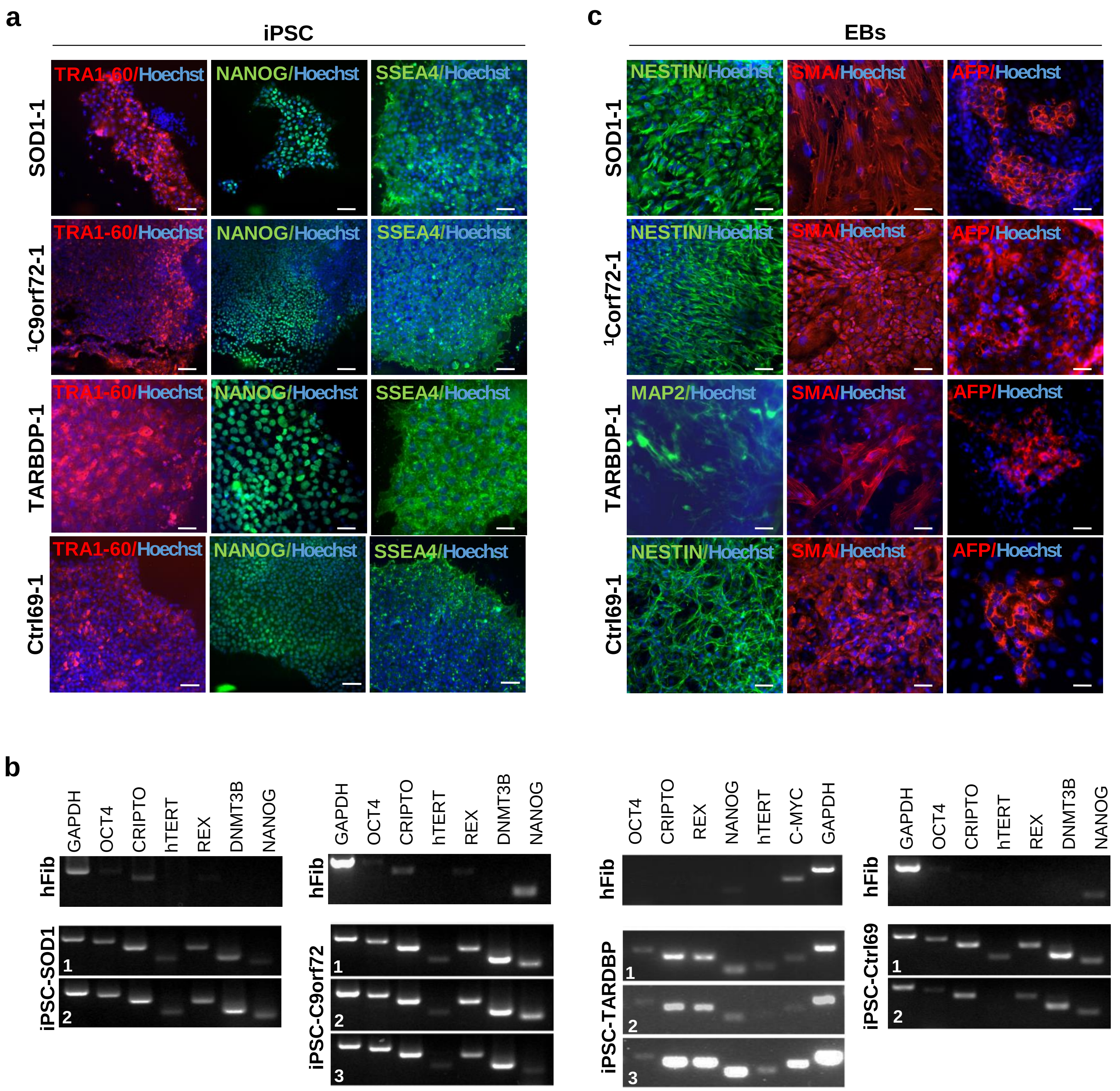

Supplementary Figure 1. Molecular and functional characterization of iPSC clones. (a) Control and ALS iPSC clones express human pluripotency-associated markers including TRA1-60, NANOG and SSEA4. Representative immuno-staining are shown for three ALS clones (SOD1-1, [1]C9ORF72-1, TARDBP-1) and one control clone (Ctrl69-1). Hoechst = H33342 labelling of nuclei. Scale bar: 100µm. (**b)** RT-PCR experiments show that all iPSC clones express RNAs encoding endogenous pluripotency-associated genes (*OCT4, CRYPTO, hTERT, REX, DNMTB3, NANOG, c-MYC*). Parental fibroblasts (hFib) are shown. *GAPDH* (glyceraldehyde-3-phosphate dehydrogenase) is the positive control. (**c)** Efficient differentiation of iPSC clones into the 3 germ layers in vitro. Embryoid Bodies (EBs) were grown for 10 days in suspension and transferred to coated coverslips for 10 more days. ALS and control EBs derived from each iPSC clone gave rise to cells of the ectoderm (labelled with antibodies directed against NESTIN or the Microtubule-Associated Protein 2 (MAP2)), the mesoderm (marked with an antibody directed against the Smooth Muscle Actin protein (SMA)) and the endoderm (marked with an antibody directed against the Alpha FetoProtein (AFP)) layers. Hoechst = H33342 labelling of nuclei. Representative images are shown. Scale bars: 50 µm.

Supplementary Fig.2

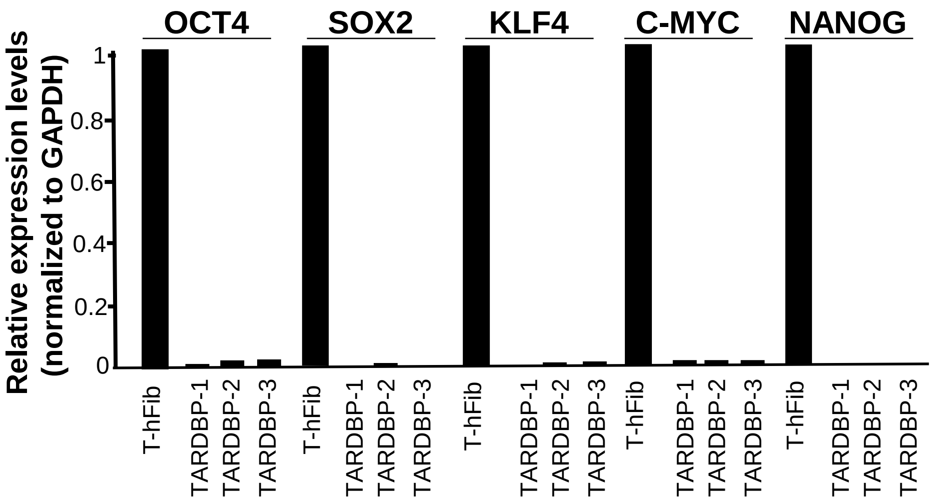

Supplementary Figure 2. Silencing of integrated retroviral vectors in TARDBP^{G348C} iPSC clones. Quantitative PCR after reverse transcription (qRT-PCR) shows that gene expression of retroviral vector genomes coding for OCT4, SOX2, KLF4, c-MYC and NANOG are silenced in iPSC clones compared to fibroblasts (T-hFib, value set to 1). Expression levels are expressed relative to the amounts of the reference GAPDH RNAs.

Supplementary Fig.3

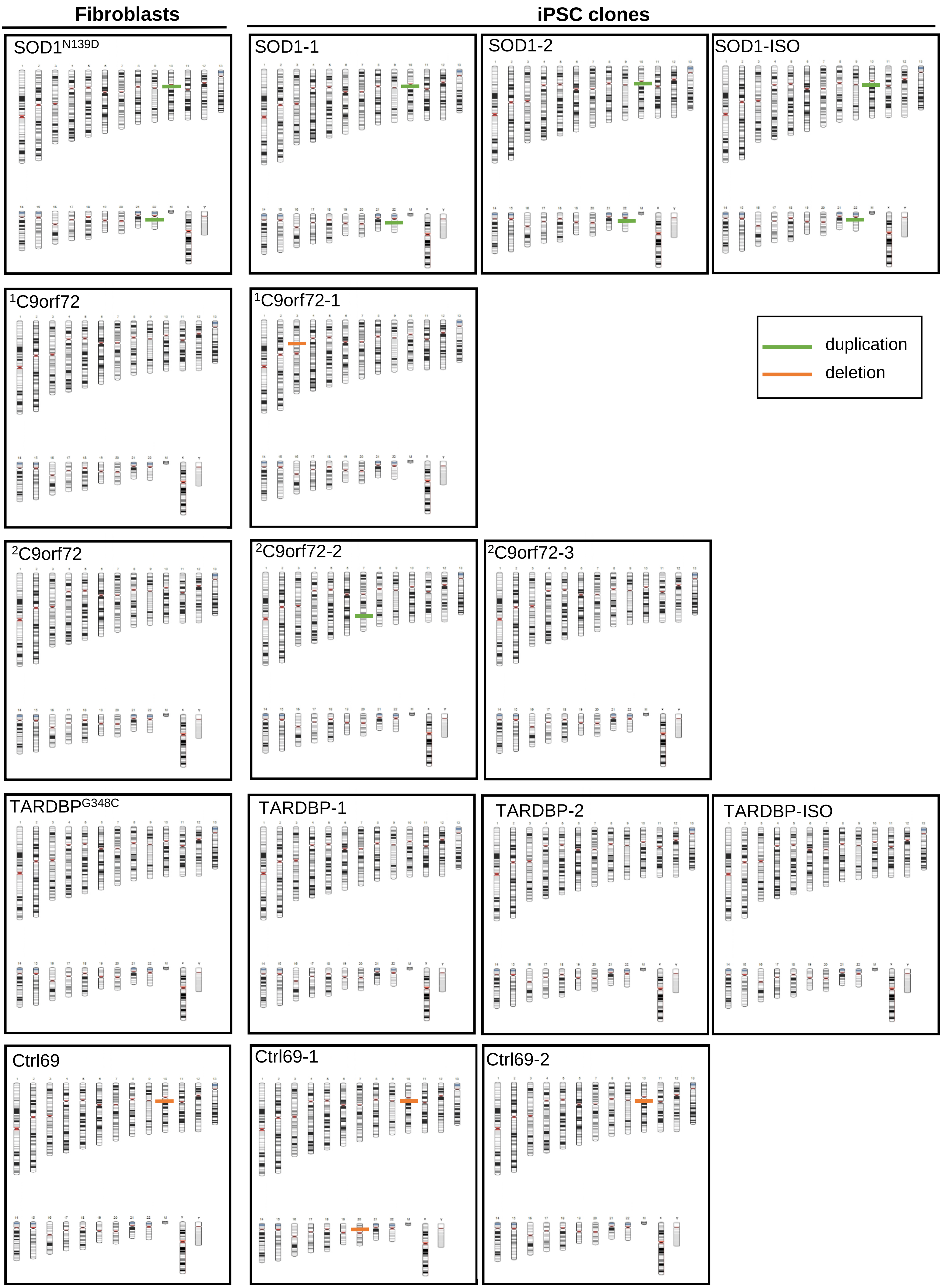

Supplementary Figure 3. Genome integrity was assessed by Illumina Human OmniExpress-24 SNP array (300,000 markers) and analyzed using KaryoStudio and GenomeStudio softwares. If SNP deviations were detected in iPSC clones, they were compared to their original pool of fibroblasts using the reference human genome. These analyzes confirmed the identity of each clone compared to its parental fibroblast and that cells had not acquired large-scale copy number variations, although a small number of small-scale indels were found (well below the level that would be detected by G-banding). Orange and green bars show small-scale indels (deletion or duplication, respectively) found in the different cell types. For some iPSC clones same anomalies were found in fibroblasts and their derivatives. For some other clones new small-scale indels were detected. Indels were compared with those reported in the Database of Genomic Variants (DGV) that contains a summary of structural variations in the human genome of a healthy control population [1]. This database allowed to show that small-scale indels found in iPSC and/or their derivatives were also present in healthy individuals and thus were most likely not associated with disease.

1. MacDonald, J.R., et al., *The Database of Genomic Variants: a curated collection of structural variation in the human genome.* Nucleic Acids Res, 2014. **42** (Database issue): p. D986-92.

Supplementary Fig.4

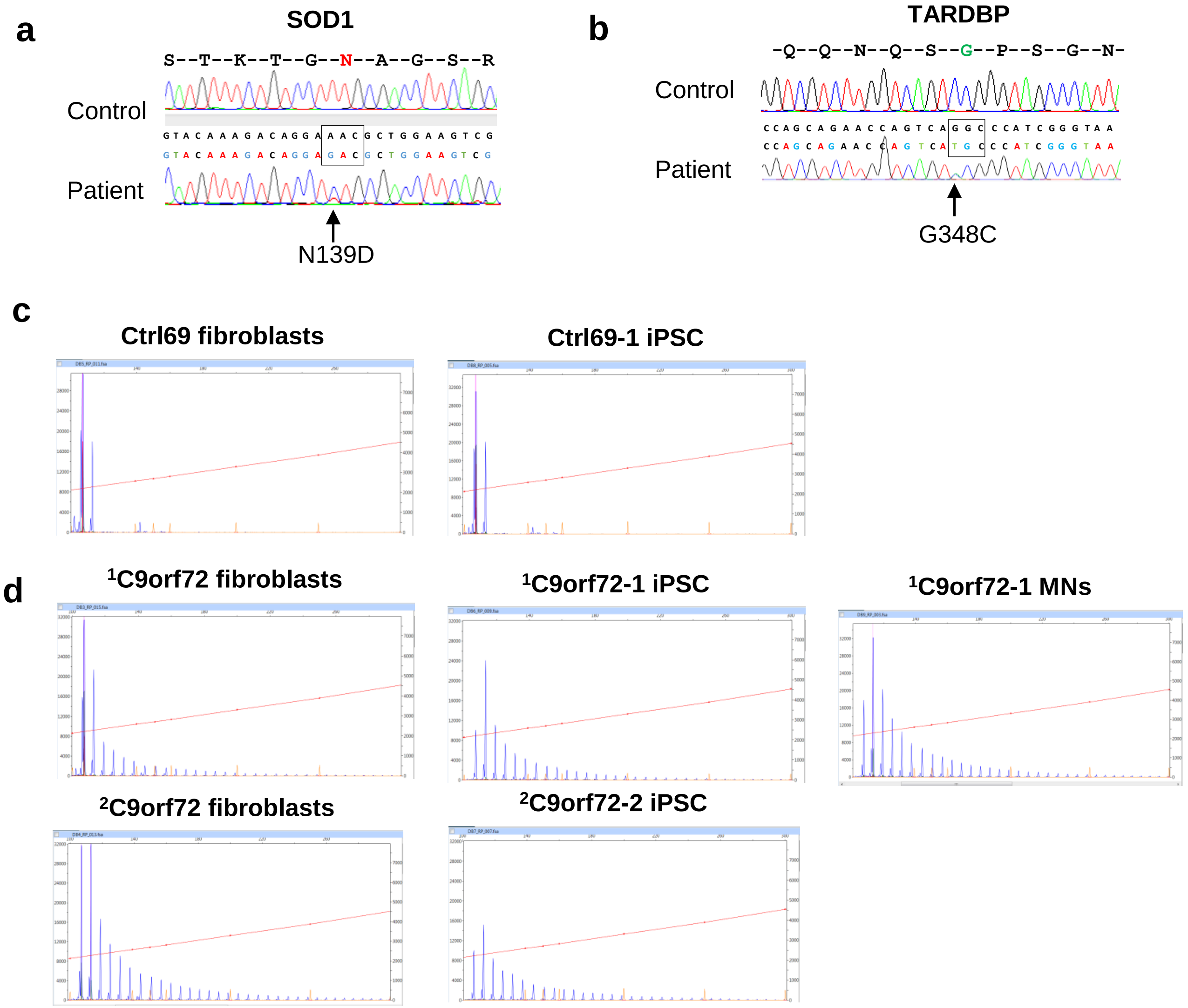

Supplementary Figure 4. Analysis of mutations in ALS patients' fibroblasts, iPSC and MNs. (a, b) DNA direct sequencing of control and patient derived-iPSC confirms the presence of (a) the heterozygous N139D mutation in the *SOD1* gene and (b) the heterozygous G348C mutation in the *TARDBP* gene in patient's cells. **(c,d)** Repeat-primed PCR was used to detect expanded GGGGCC hexanucleotides. The reverse primer designed on the repeat sequence allowed the amplification of multiple fluorescent PCR products that were visualized using GeneMapper software v4.0. Profiles are shown for (c) parental Ctrl69 fibroblasts and the Ctrl69-1 iPSC clone, (d) fibroblasts from [1]C9orf72 and [2]C9orf72 patients, the [1]C9orf72-1 and [2]C9orf72-2 iPSC clones, and MNs derived from [1]C9orf72-1 iPSC clone.

Supplementary Figure 5

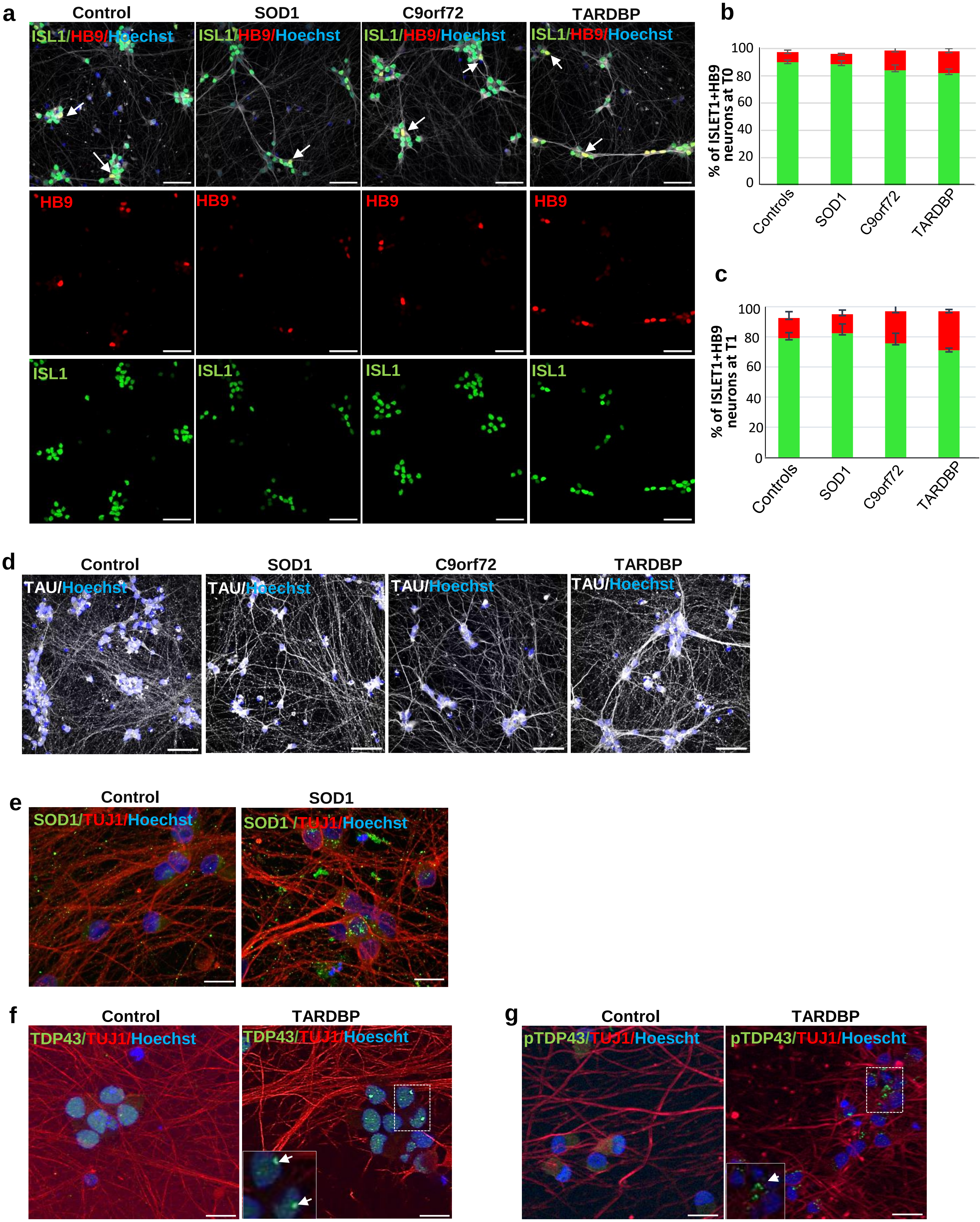

Supplementary Figure 5. Characterization of iPSC-derived MNs and protein accumulations.

(a-c) Expression of HB9 and ISLET1 in control, SOD1, C9orf72 and TARDBP MNs. (a) Representative images of β3-tubulin-positive (TUJ1) neurons co-expressing HB9 (in red) and ISLET1 (in green) are shown. Nuclei are stained with Hoescht H33342. Scale bars: 20µm. (b, c) Graph show percentages of neurons co-expressing HB9 and ISLET1 at T0 (c) and T1 (d). All HB9-positive cells were also ISLET1 positive. Mean percentages were 10.9 ±1.5 % of HB9-ISLET1-double positive MNs, at T0 and 18 ± 2.3 % of HB9-ISLET1-double positive MNs at T1 (mean ± SEM). At T0: 2 independent experiments with 3 control clones (Ctrl40, n=1, Ctrl60, n=1, Ctrl69-1, n=1), and SOD1-2 (n=2), [1]C9orf72-1 (n=2) and TARDBP-1 (n=2) clones were analyzed. At T1: 3 independent experiments with 4 control clones (Ctrl40, n=1, Ctrl60, n=1, Ctrl69-1, n=2), and SOD1-2 (n=3), [1]C9orf72-1 (n=2), [2]C9orf72-2 (n=1), and TARDBP-1 (n=3) clones were analyzed.

(d) Expression of TAU in control, SOD1, C9orf72 and TARDBP mMNs. Representative images of TAU-positive neurons (in red) are shown. Nuclei are stained with Hoescht H33342. Scale bars: 20µm.

(e) Accumulation of misfolded SOD1 in $SOD1^{N139D}$ MNs. Representative images show control and SOD1 mMNs labelled with antibodies directed against a misfolded form of SOD1 (C4F6 antibody, in green) and β3-tubulin (TUJ1, in red). Nuclei are stained with Hoescht H33342. Scale bars: 20µm.

(f, g) Very rare (e) TDP-43 and (f) phospho-TDP43 accumulations were observed in $TARDBP^{G348C}$ mMNs. Representative images show control and TARDBP mMNs co-labelled with antibodies directed against either TDP-43 (in green) or phosphoTDP-43 (in green), and β3-tubulin (TUJ1, in red). Nuclei are stained with Hoescht H33342. Scale bars: 20µm.

Supplementary Fig.6

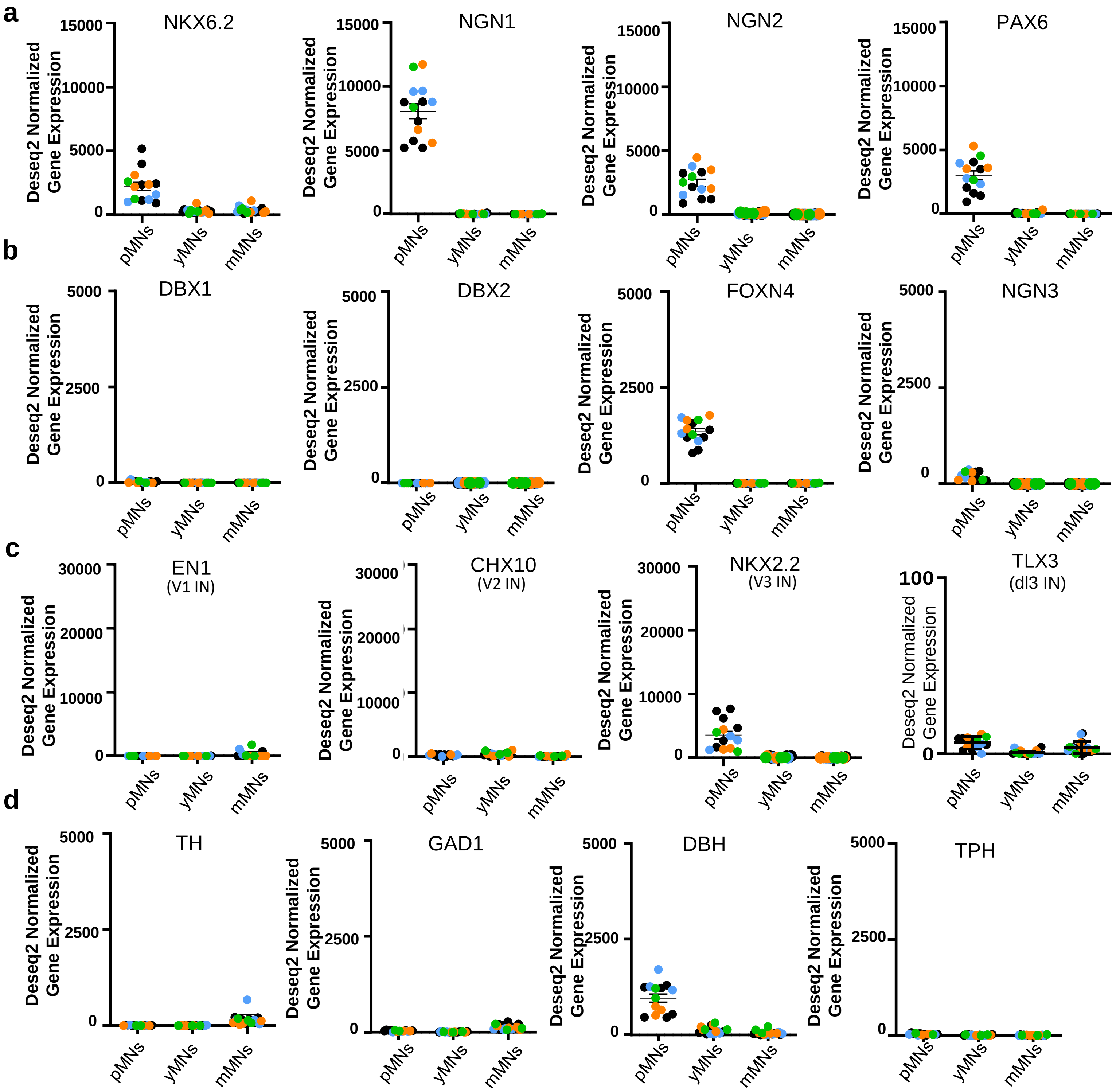

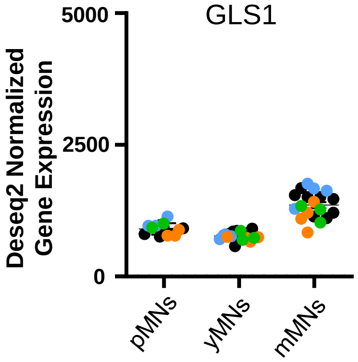

Enzyme	Neurone type
TH	Dopaminergic
GAD1	GABAergic
TPH	Serotoninergic
DBH	Noradrenergic
GLS1	Glutamatergic

Supplementary Fig.6

e

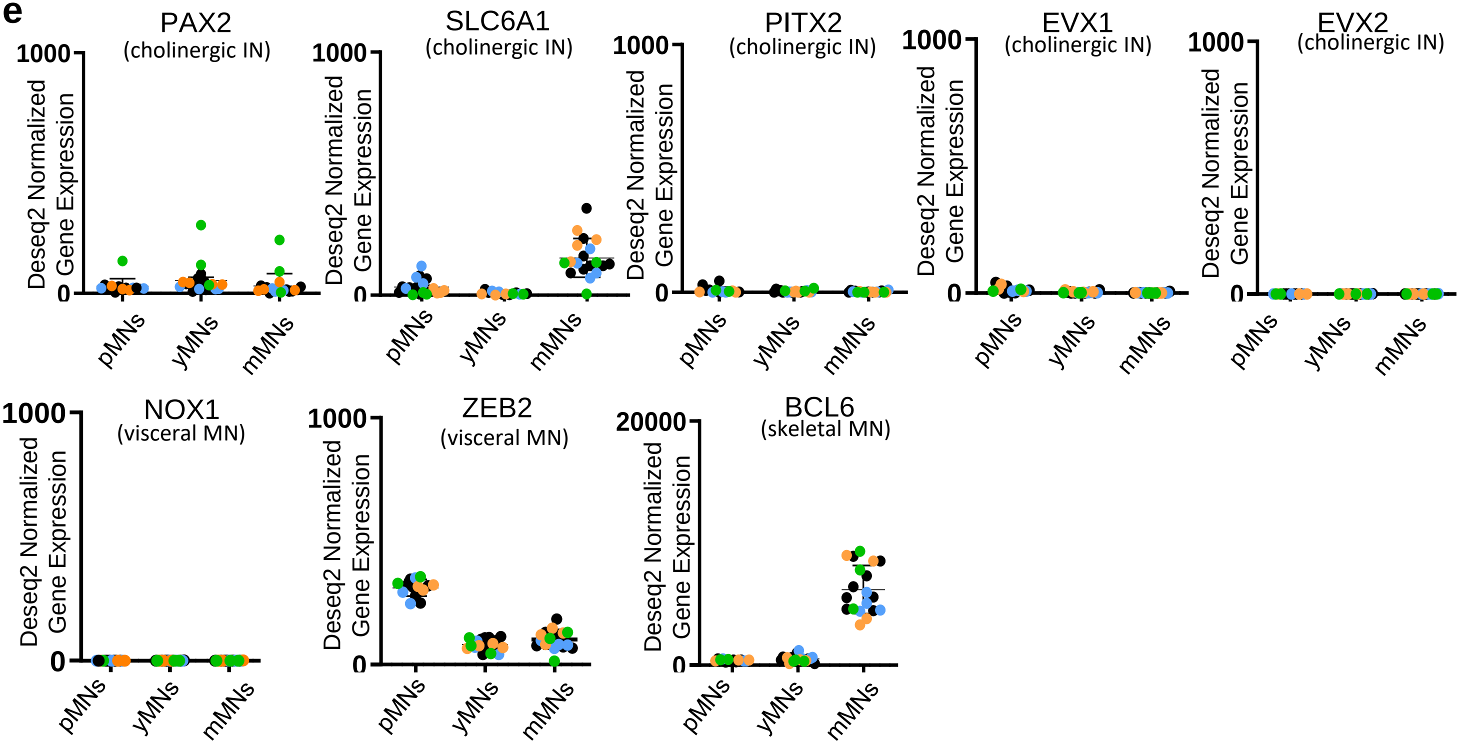

f

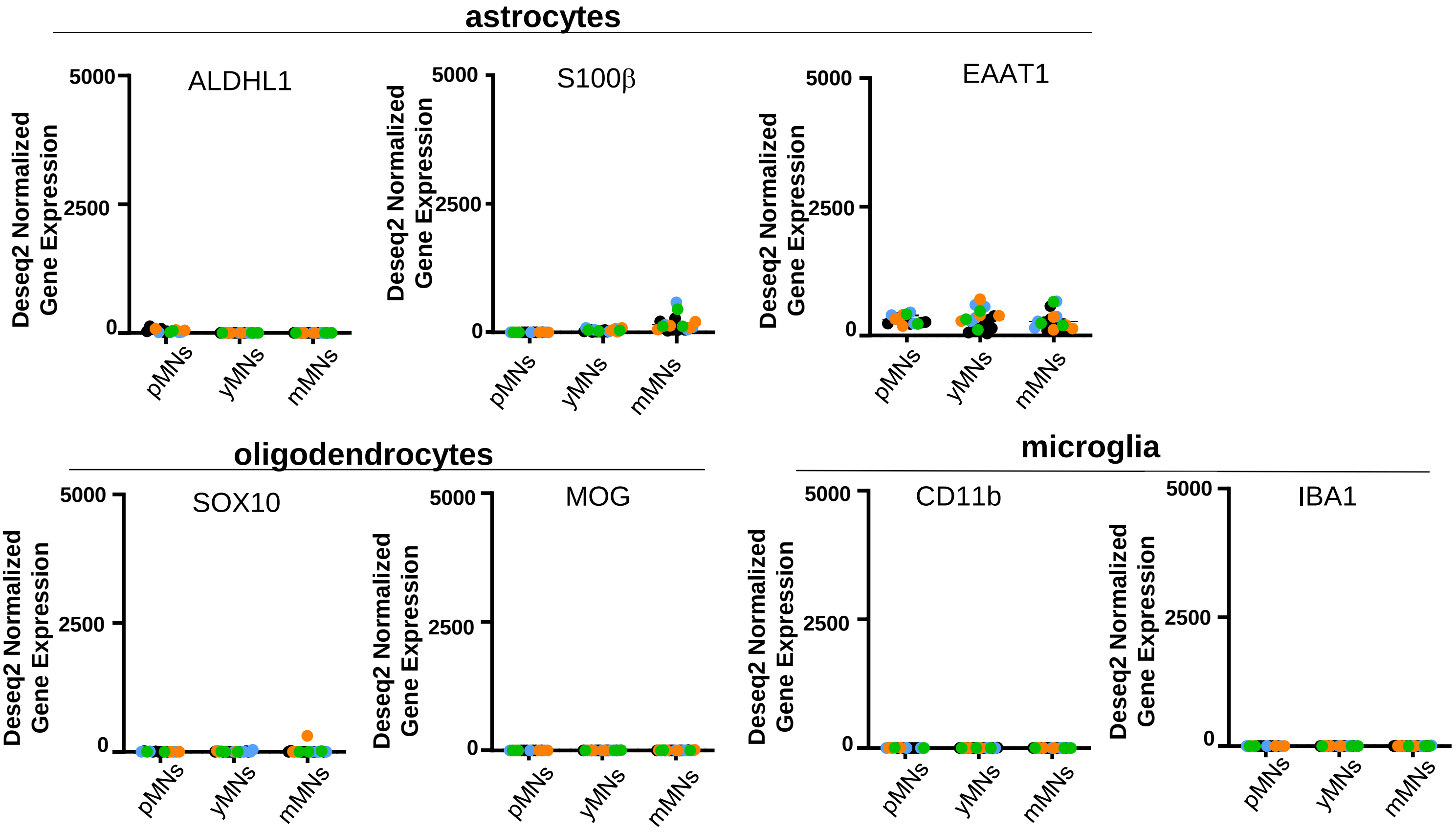

g

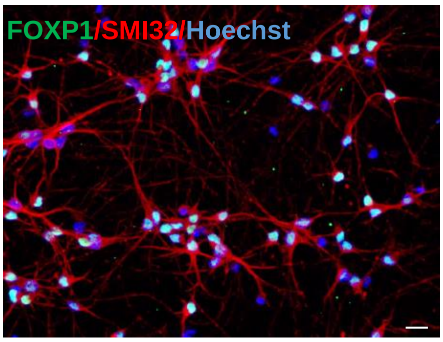

h

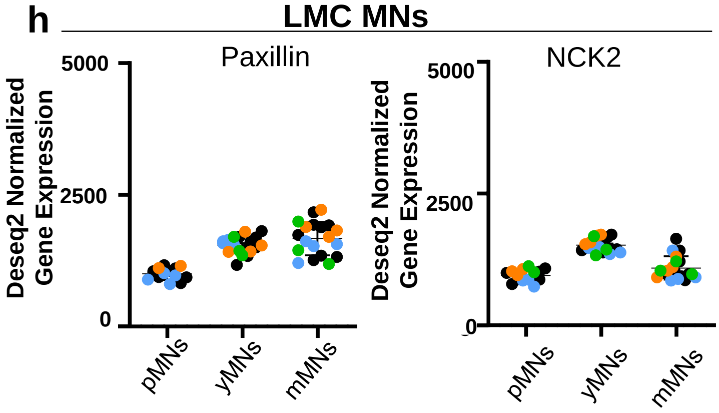

Supplementary Fig.6

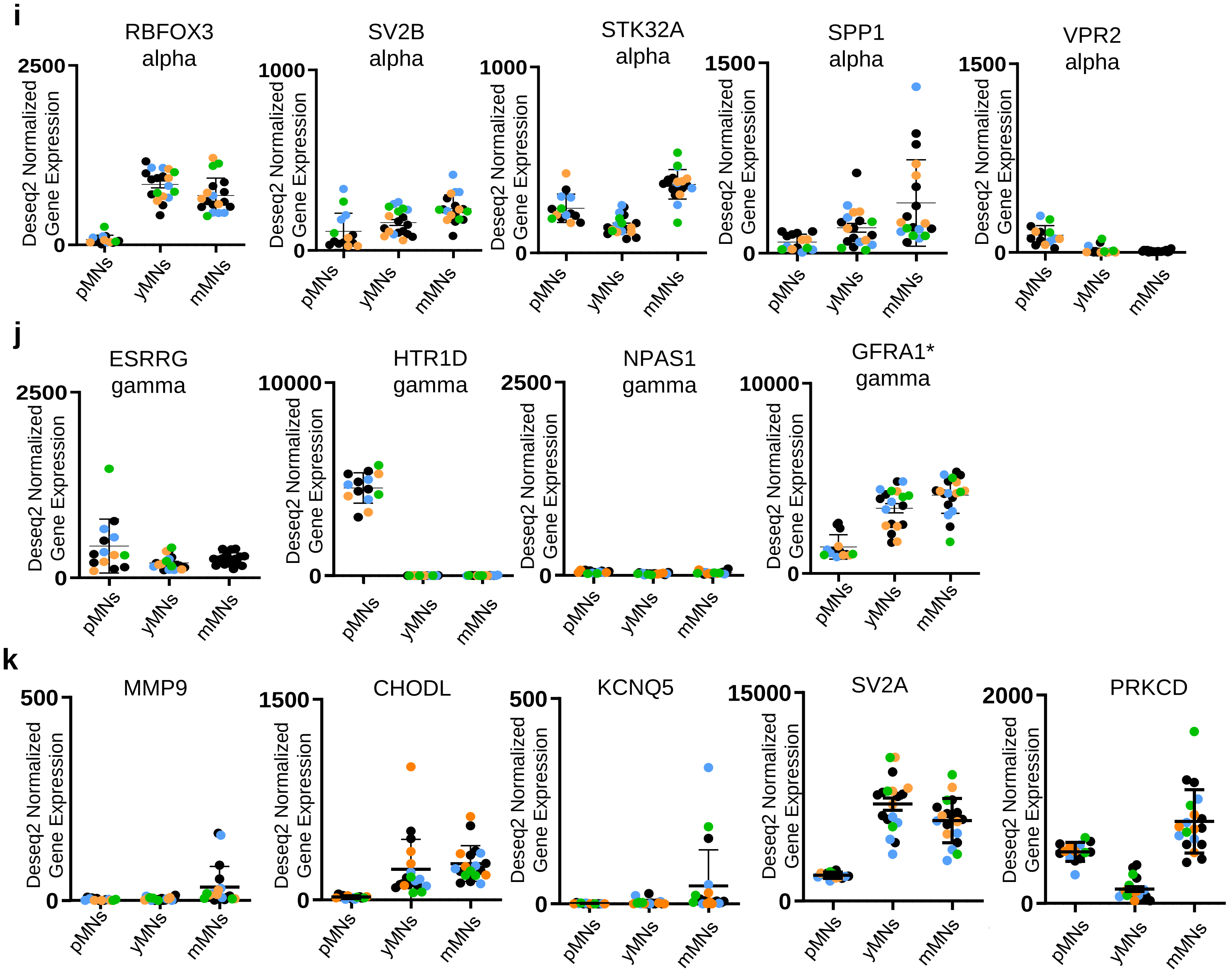

Supplementary Figure 6. DeSeq2 normalized gene expression of genes, including transcription factors and enzymes, in pMNs, yMNs and mMNs. (Additional data to Fig 2. Black dots for control MNs. Blue dots for SOD1 MNs. Orange dots for C9orf72 MNs. Green dots for TARDBP MNs). **(a-g)** (a) *NKX6.2, NGN1, NGN2, PAX6.* (b) *DBX1, DBX2, FOXN4, NGN3.* (c) Engrailed 1 (*En1*), *CHX10*, *NKX2.2, TLX3*. (d) Tyrosine Hydroxylase (*TH*), Glutamate Decarboxylase (*GAD1*), Dopamine β-Hydroxylase (*DBH*), Tryptophan Hydroxylase (*TPH*), *and* Glutaminase (*GLS1*). The table lists the different enzymes and neuron subtypes expressing these enzymes. (e) *PAX2, SLC6A1, PITX2, EVX1, EVX2* (for cholinergic interneurons), *NOX1, ZEB2* (for visceral MNs), *BCL6* (for skeletal MNs). Expression of *HNF6*, reported to be expressed in both cholinergic interneurons and visceral MNs was not detected. (f) Aldehyde dehydrogenase 1 (*ALDHL1*), *S100b*, Excitatory Amino Acid Transporter 1 (*EAAT1*), *SOX10*, Myelin Oligodendrocyte protein (*MOG*), *CD11b and IBA1*. (g) *Paxillin* and *NCK2*. (**h**) Representative immuno-staining images show mMNs expressing FOXP1 (in green) and SMI32 (in red). Nuclei were stained with Hoescht H33342. Scale bar: 50 µm. **(i, j)** *RBFOX3, SV2B, STK32A, SPP1, VPR2* (alpha-MN markers), *ESRRG, HTR1D, NPAS1, GFRA1** (gamma-MN markers). Only the expression of *GFRA1* increased in yMNs and mMNs but it remains difficult to conclude if we have gamma-MNs in our cultures because (1) this is the only gamma marker expressed in mMNs and (2) GFRA1 expression is dose-dependent in MNs subtypes. **(k)** *MMP9*, *CHODL*, *KCNQ5* for fast-fatigable (FF) and fast-fatigable resistant (FR) MNs. *SV2A* and *PRKCD* for slow firing (FS) MNs.

Supplementary Fig.7

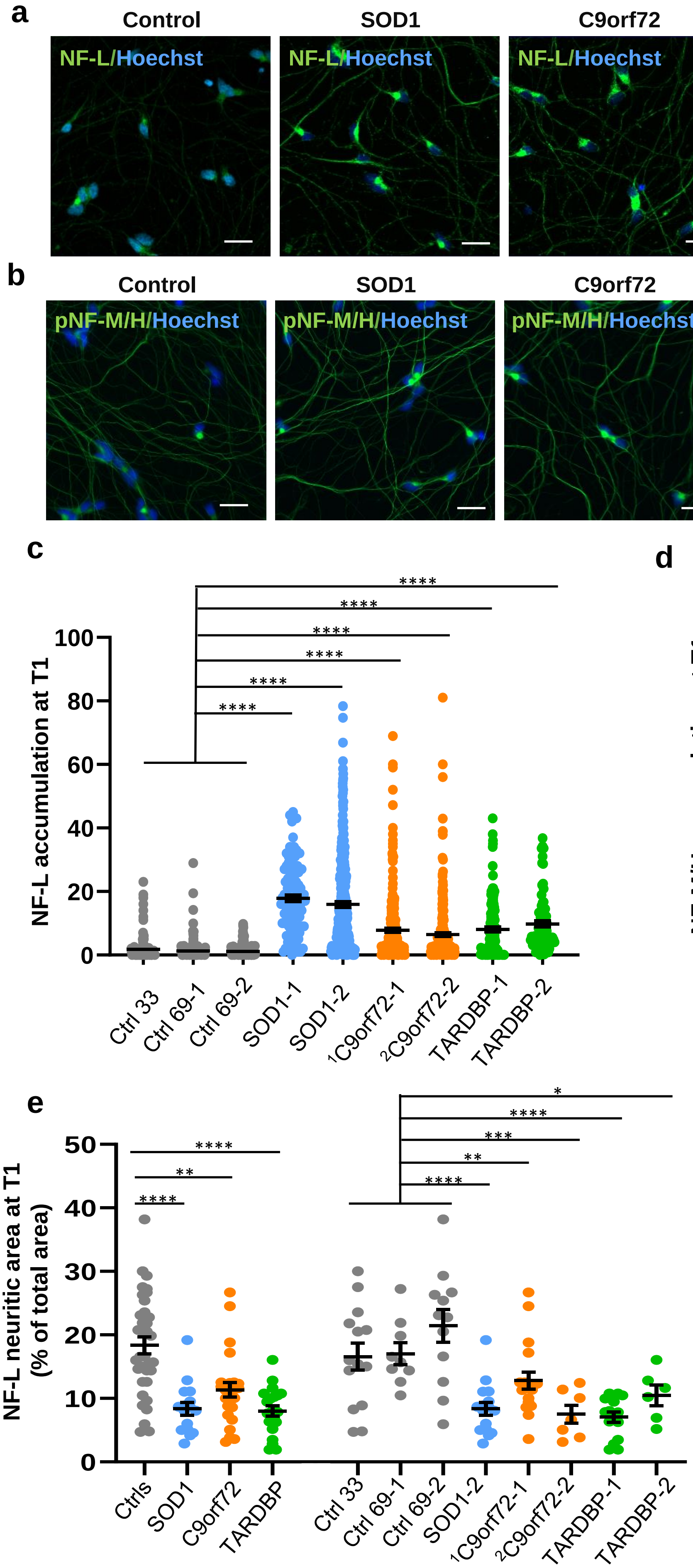

Supplementary Figure 7. (**Additional data to Fig. 3**). (a, b) NF accumulation in ALS yMNs (at T0). Images show representative cultures of yMNs that were immunolabelled with antibodies directed against NF-L (a) or pNF-M/H (b). In these MNs NF-L and pNF-M/H soma accumulations were more pronounced in SOD1 and C9orf72 MNs than in control MNs and TARDBP MNs. Nucleus were stained with Hoechst H33432. Scale bars: 10µm. (c) Quantifications of soma NF-L signal intensities in MNs in individual iPSC clones at T1. n = number of analyzed neurons for each clone. Statistical significance by Kruskal-Wallis with Dunn's test (****p<0.0001). Ctrl33: n = 178; Ctrl69-1: n = 174; Ctrl69-2: n = 225; SOD1-1: n = 106; SOD1-2: n = 316; [1]C9orf72-1: n = 297; [2]C9orf72-2: n = 269; TARDBP-1: n = 135; TARDBP-2: n = 79. (d) Quantification of pNF-M/H accumulations at T1 in individual iPSC clones. Each dot represents the percentage of neurons with pNF-M/H accumulations counted in one experiment. Mean ± SEM. Statistical significance by unpaired t-test (**p<0.01; ****p<0.0001). Ctrl33: n = 3; Ctrl69-1: n = 2; Ctrl69-2: n = 2; SOD1-1: n = 3; SOD1-2: n = 3; [1]C9orf72-1: n = 3; [2]C9orf72-2: n = 3; TARDBP-1: n = 3; TARDBP-2: n = 3. (e) Quantification of NF-L signal intensities only in neurites of control and ALS MNs at T1. Values are percentages of area occupied by all positive neurite signals per total area. One area corresponds to an image of 37533µm^2. n = number of analyzed images in 7 independent experiments (see Methods). Both pooled and individual data are shown. No statistical difference was found between the different controls. Mean ± SEM. Statistical significance by Kruskal-Wallis with Dunn's test (* p<0.05; **p<0.01; ***p<0.001; ****p<0.0001). Ctrl33: n= 14; Ctrl69-1: n= 9; Ctrl69-2: n= 12; SOD1-2: n=16; [1]C9orf72-1: n=18; [2]C9orf72-2: n=7; TARDBP-1: n=15 ;TARDBP-3: n=6.

Supplementary Fig.8

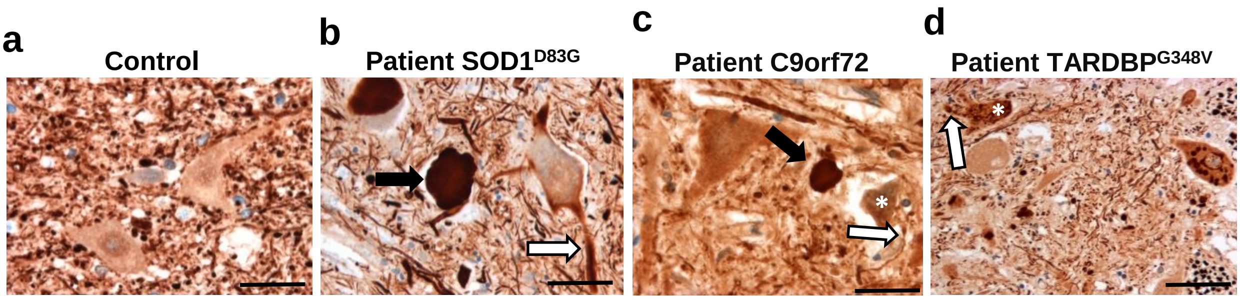

Supplementary Figure 8. Neurofilament accumulations in MNs of ALS patients. High magnification images of human spinal cord sections show pNF-H staining (brown) and Harris hematoxylin counterstaining (blue) in (a) a control subject and (b-d) 3 ALS patient's tissues. Images show spinal cord anterior horns. The patients carry either the $SOD1^{D83G}$ mutated gene, or expansions in the C9orf72 gene, or the $TARDBP^{G348V}$ mutated gene. In the spinal cord section of the control subject, the pNF-H staining shows very thin neurite fragments and some small positive dots around 2 normal MNs. In ALS spinal cord sections, the pNF-H staining shows a less dense neurite network and two patterns of abnormal pNF-H accumulations. Black arrows show large axonal beads and white arrows show accumulations in neurites proximal to soma. Shrinked MNs can also be observed in ALS tissues (stars). Scale bars: 40µm.

Supplementary Fig.9

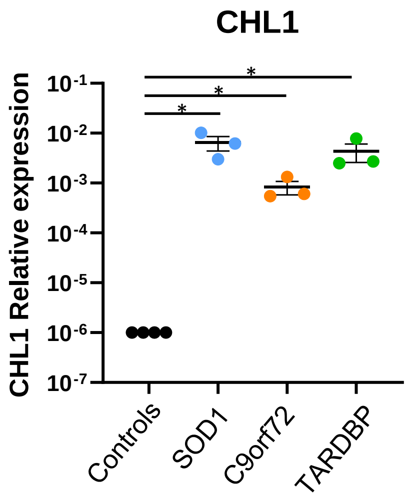

Supplementary Figure 9. Higher RNA expression levels of CHL1 in ALS MNs. Quantitative PCR after reverse transcription (qRT-PCR) assessed RNA levels of the Cell adhesion molecule L1 like protein (CHL1) in MNs derived from control (n=4), $SOD1^{N139D}$, C9orf72 and $TARDBP^{G348C}$ MNs (n=3 for each). Expression levels are expressed relative to the amounts of the reference GAPDH RNAs. Statistical significance by unpaired t-test (*p <0.05).

Supplementary Fig. 10

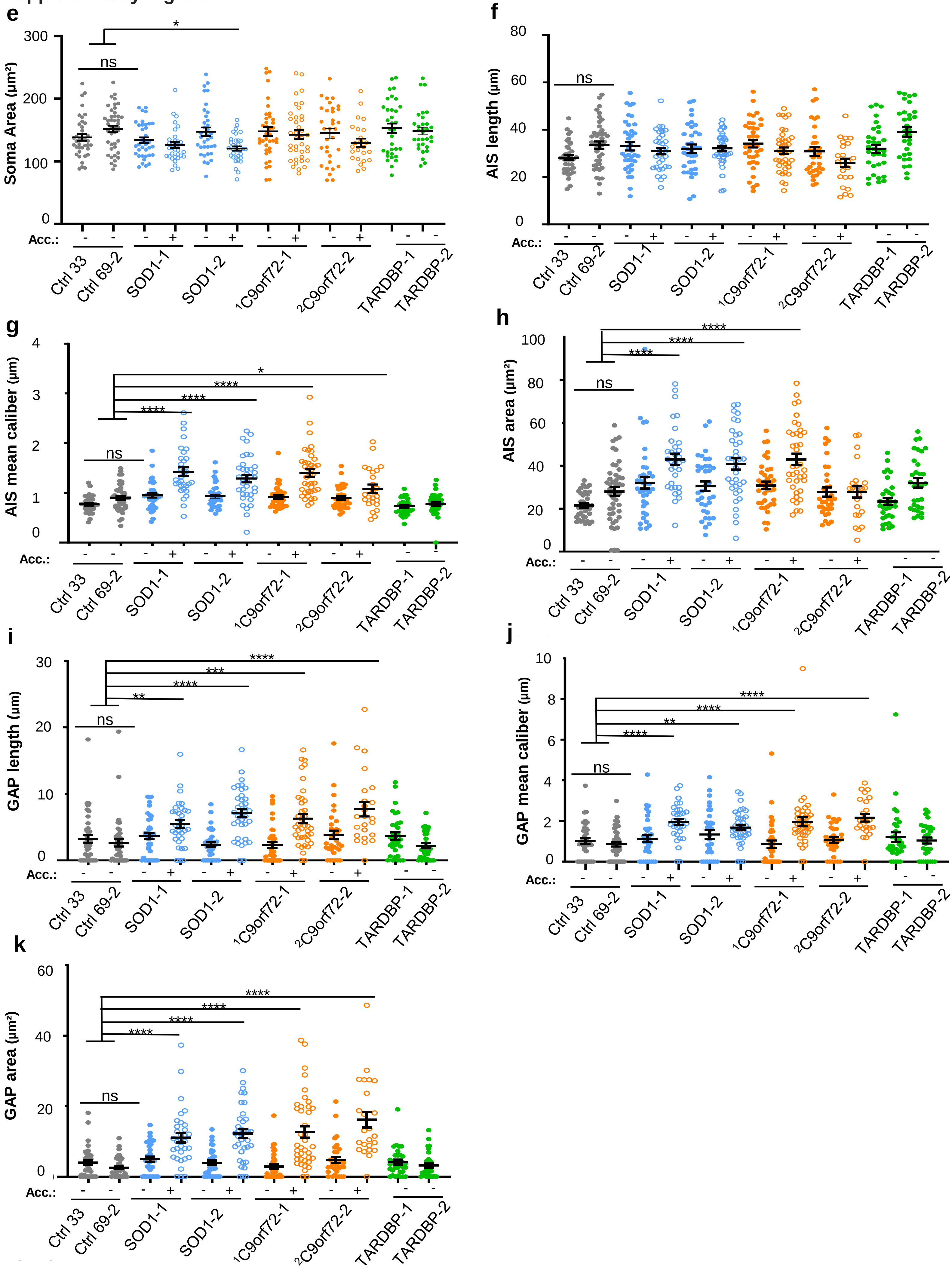

Supplementary Figure 10e-k (Additional data to Figure 4e-k). Graphs show quantifications done on images acquired on MN cultures derived from each iPSC clone: Ctrl33, Ctrl69-2, SOD1-1, SOD1-2, [1]C9orf72-1, [2]C9orf72-2, TARDBP-1 and TARDBP-2, with or without pNF-M/H accumulations (Acc). (e) soma areas. (f-k) length, mean axonal caliber and area of AIS (f, g, h) and GAP (i, j, k). Statistical significance shown are between pooled controls MNs (no statistical difference between both controls) and each one of the ALS MNs with or without accumulation by Kruskal-Wallis with Dunn's test (mean $\pm$ SEM). (*$p<0.05$, **$p<0.01$, ***$p<0.001$, ****$p<0.0001$). Ctrl33: n = 37; Ctrl69-2: n = 44; SOD1-1: n = 37 (with acc.) / n = 33 (without acc.); , SOD1-2: n = 35 (with acc.) / n =35 (without acc.); [1]C9orf72-1: n = 30 (with acc.) / n = 38 (without acc.). [2]C9orf72-2): n = 33 (with acc.) / n = 23 (without acc.). TARDBP-1: n = : 33; TARDBP-2: n = 31.

Supplementary Fig. 11

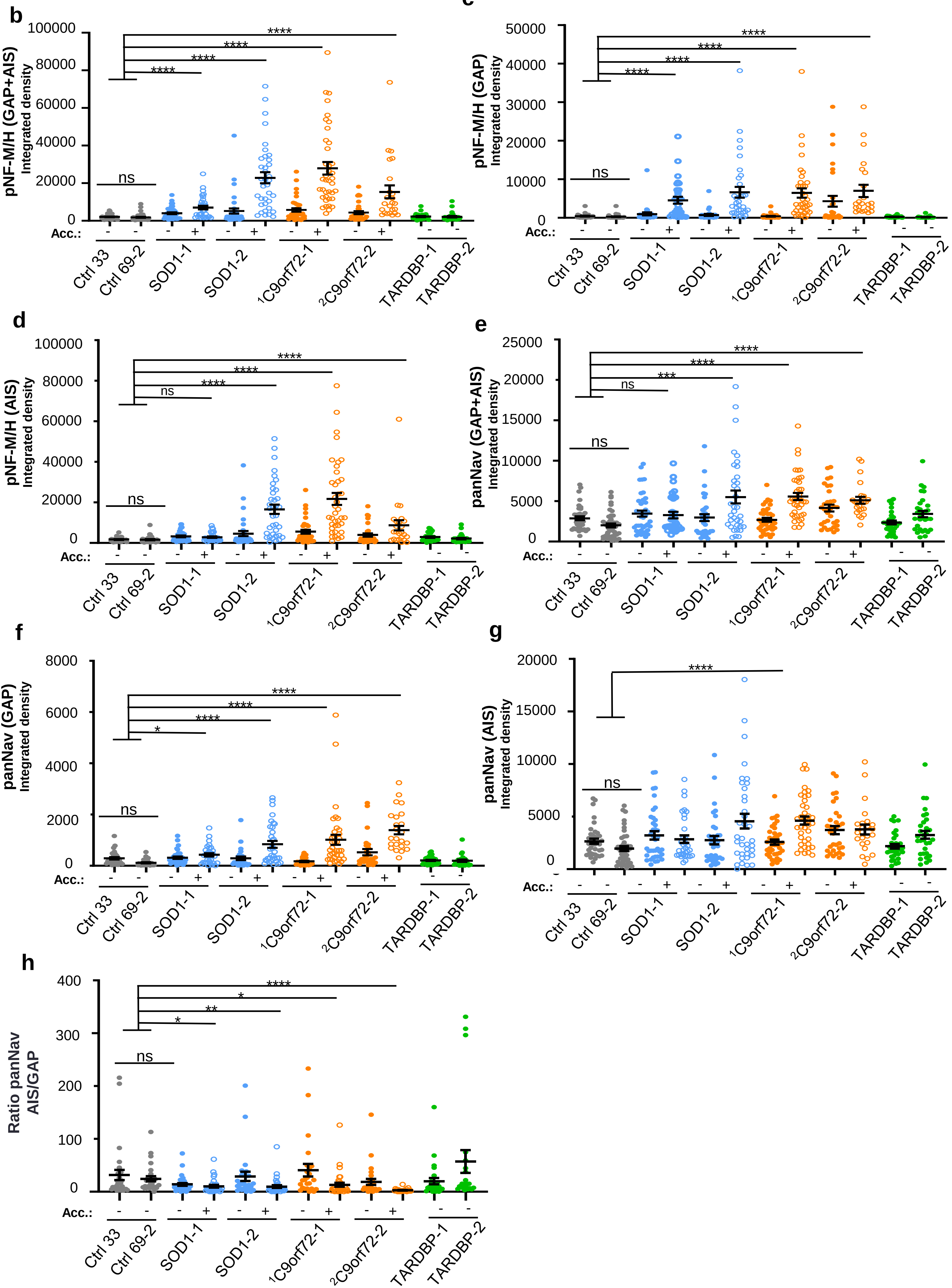

Supplementary Figure 11b-h (Additional data to Figure 5b-h). Integrated densities of pNF-M/H and panNav signals were calculated to assess their distributions in the GAP and the AIS in MN cultures derived from each iPSC clone: Ctrl33, Ctrl69-2, SOD1-1, SOD1-2, [1]C9orf72-1, [2]C9orf72-2, TARDBP-1 and TARDBP-2, with or without pNF-M/H accumulations (Acc). (b-d) pNF-M/H integrated density measured (b) between the soma and the end of the AIS (GAP+AIS), (c) in the GAP and (d) in the AIS. (e-g) PanNav integrated density measured (e) between the soma and the end of the AIS (GAP+AIS), (f) in the GAP and (g) in the AIS. (h) Ratio of panNav integrated density in the AIS to the GAP. Statistical significance shown are between pooled controls MNs (no statistical difference between both controls) and each one of the ALS MNs with or without accumulation by Kruskal-Wallis with Dunn's test (mean ± SEM). (*p<0.05, **p<0.01, ***p<0.001, ****p<0.0001). Ctrl33: n = 30; Ctrl69-2: n =29; SOD1-1: n = 34 (with acc.) / n = 30 (without acc.); , SOD1-2: n = 26 (with acc.) / n = 34 (without acc.); [1]C9orf72-1: n =24 (with acc.) / n = 37 (without acc.). [2]C9orf72-2): n = 27 (with acc.) / n = 24 (without acc.). TARDBP-1: n = 28 ; TARDBP-2: n = 23.

Supplementary Fig.12

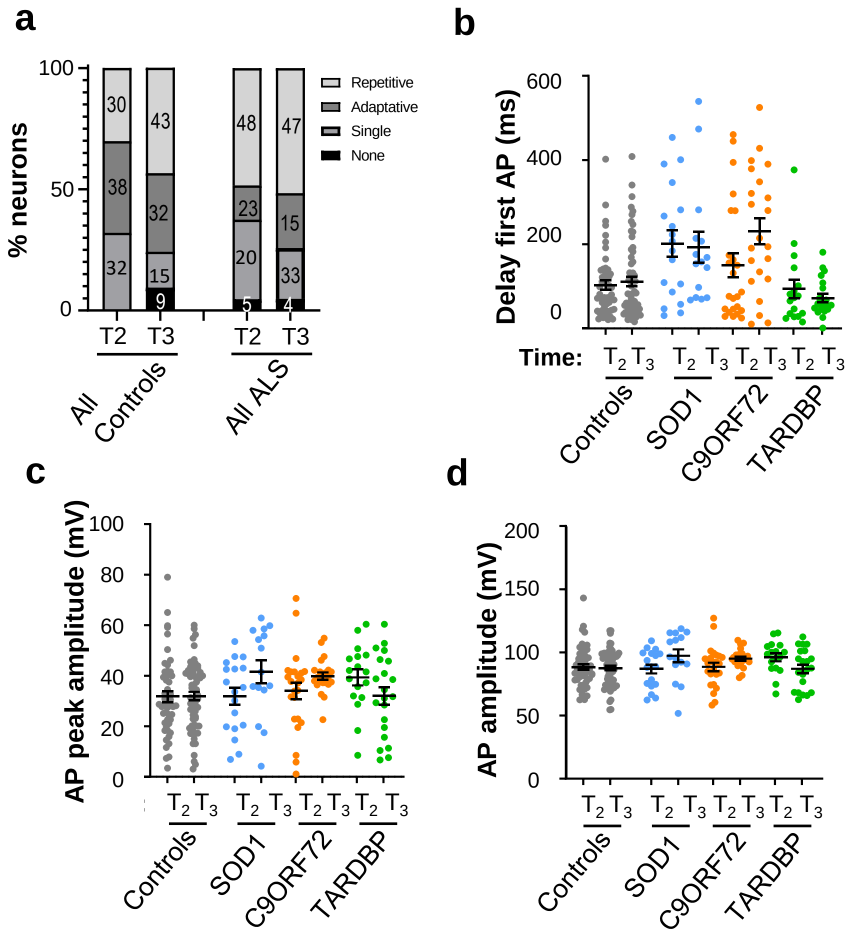

Supplementary Figure 12. Additional parameters of current-clamp recordings. (Additional data to Fig 6). **(a)** Whole-cell patch-clamp recordings of iPSC-derived MNs from control subjects and from all ALS patients at T2 and T3. See Figure 6 for numbers of analyzed neurons. No significant difference was measured between control and ALS MNs. **(b)** Delay to the first peak, **(c)** peak amplitude, **(d)** Action Potential (AP) amplitude measured at T2 and T3 in control and mutant iPSC-derived MNs.
