# Supplementary material for: Neurofilament accumulations in amyotrophic lateral sclerosis patients’ motor neurons impair axonal initial segment integrity
Source: Cell Mol Life Sci. 2023 May 15;80(6):150. doi: 10.1007/s00018-023-04797-6 (PMC10185656; doi:10.1007/s00018-023-04797-6)
Supplement: Supplementary file 4 — Supplementary file4 (DOCX 46 KB) [file 18_2023_4797_MOESM4_ESM.docx]

**Supplementary Table 1:** Description of ALS patients (*post-mortem* tissues).

| ALS gene / mutation | Sex | Age at death | Progression |
| --- | --- | --- | --- |
| SOD1^D83G^ | male | 73 | < 1year |
| C9orf72-HRE | male | 55 | 1.6 years |
| TARDBP^G348V^ | male | 47 | 3 years |

HRE= hexanucleotides repeat expansions.

**Supplementary Table 2:** Primary antibodies.

| Primary Antibodies | Isotype | Dilution | Supplier |
| --- | --- | --- | --- |
| AFP | IgG2a | 1/200 | Chemicon |
| AnkG | IgG2a | 1/200 | Neuromabs |
| FOXP1 | Rabbit | 1/200 | Abcam |
| HB9 * | IgG1 | 1/50 | DSHB |
| ISLET1 | Rabbit | 1/100 | Abcam |
| MAP2 | Rabbit | 1/500 | Synaptic System |
| NANOG | Rabbit | 1/100 | Abcam |
| NESTIN | Rabbit | 1/500 | Millipore |
| NF-L | Igg1 | 1/200 | Sigma |
| OLIG2 | Rabbit | 1/1000 | Millipore |
| Pan-Nav | Rabbit | 1/200 | Alomone Labs |
| p-NFM/H | Igg1 | 1/300 | Millipore |
| SMA | IgG2a | 1/200 | Chemicon |
| SMI-31 | IgG1 | 1:4000 | Biolegend |
| SOD1 | IgG1 | 1/100 | Medimabs |
| SSEA-4 | IgG3 | 1/100 | Chemicon |
| SMI-32 | IgG | 1/1000 | Covance |
| TAU | Rabbit | 1/200 | Sigma |
| TDP-43 | Rabbit | 1/100 | ProteinTech |
| pTDP-43 | IgG1 | 1/3000 | CosmoBio |
| TRA1-60 | IgGM | 1/100 | Chemicon |
| Tuj1 | Igg2a | 1/500 | Covance |
| VACHT | Rabbit | 1/1000 | Synaptic Systems |

* 81.5C10 was deposited to the DSHB by Jessell, T.M. / Brenner-Morton, S. (DSHB Hybridoma Product 81.5C10)

**Supplementary Table 3:** Fluorescent secondary antibodies

| Secondary Antibodies | Dilution | Supplier |
| --- | --- | --- |
| goat anti-mouse IgG1 AlexaFluor® 488 | 1/2000 | Molecular Probes |
| goat anti-mouse IgG1 AlexaFluor® 555 | 1/2000 | Molecular Probes |
| goat anti-mouse IgG1 AlexaFluor® 647 | 1/500 | Molecular Probes |
| goat anti-mouse IgG2a AlexaFluor® 488 | 1/500 | Molecular Probes |
| goat anti-mouse IgG2a AlexaFluor® 555 | 1/500 | Molecular Probes |
| goat anti-mouse IgG2a AlexaFluor® 647 | 1/500 | Molecular Probes |
| goat anti-rabbit AlexaFluor® 647 | 1/500 | Jackson ImmunoResearch |
| goat anti-rabbit AlexaFluor® 488 | 1/1000 | Molecular Probes |
| goat anti-rabbit AlexaFluor® 555 | 1/1000 | Molecular Probes |

**Supplementary Table 4.** ALS patients, control subjects and iPSC clones.

| ALS gene / mutation | Clones | Sex | Age at biopsy | Duration |
| --- | --- | --- | --- | --- |
| SOD1^N139D^ | SOD1-1  SOD1-2  SOD1-3  SOD1-ISO | woman | 50 | 8 years |
| ^1^C9orf72 | ^1^C9ORF72-1 | man | 70 | 3 years |
| ^2^C9orf72 | ^2^C9orf72-2  ^2^C9orf72-3 | man | 51 | 3 years |
| TARDBP^G348C^ | TARDBP-1 TARDBP-2  TARDBP-3  TARDBP-ISO | man | 53 | 6 years |
| Control Ctrl33 | Ctrl33 | man | 33 | - |
| Control Ctrl40 | Ctrl40 | woman | 40 | - |
| Control Ctrl60 | Ctrl60 | man | 60 | - |
| Control Ctrl69 | Ctrl69-1  Ctrl69-2 | woman | 69 | - |

ISO = isogenic control iPSC clone generated with the CRISPR/CAS9 technology.

**Supplementary Table 5: Excel file**

The excel file shows results of comparisons between yMNs and mMNs in each mutant versus control MNs. The file tabs correspond to the results for each patient. Analysis of these data and of deregulated genes are shown in Supplemental table 6 (for SOD1), table 7 (for C9orf72) and table 8 (for TARDBP).

**Supplementaty Table 6.**

Gene deregulated with time between yMNs and mMNs in SOD1 MNs versus control MNs (fold-change ≥ 1.5; adjusted P-Value ≤ 0.05). Among deregulated genes, ENC1 encodes for a protein associated to actin filaments which are crucial at the AIS. ENC1 was also shown to modulate huntingtin aggregation through p62 [1]. Another interesting gene is ST3GAL6 coding for an enzyme of the sialyltransferase family and which is a gene recently suggested to be mutated in a new atypical fALS family [2].

| **Gene symbol** | **Gene Name** | **Fold Change** | **Adjusted**  **P-Value** |
| --- | --- | --- | --- |
| **ENC1** | ectodermal-neural cortex 1 | - 8.79 | 3.71x10^-5^ |
| **UBL3** | Ubiquitin like 3 | 2.35 | 4.57x10^-3^ |
| **FASN** | Fatty acid synthase | - 1.55 | 5.02x10^-3^ |
| **ST3GAL6** | ST3 beta-galactoside alpha-2,3-sialyltransferase 6 | 2.01 | 5.83x10^-3^ |
| **RIMBP2** | RIMS binding protein 2 | - 4.54 | 2.76x10^-2^ |
| **CISH** | Cytokine inducible SH2 containing protein | - 2.17 | 2.90x10^-2^ |
| **NTN4** | netrin 4 | - 1.75 | 2.78x10^-2^ |
| **NACC2** | NACC family member 2 | - 1.88 | 3.00x10^-2^ |
| **PDZRN4** | PDZ domain containing ring finger 4 | - 4.54 | 3.56x10^-2^ |
| **MEIS** | Meis homeobox 1 | 1.57 | 3.71x10^-2^ |
| **RAMP1** | receptor activity modifying protein 1 | 3.04 | 4.31x10^-2^ |

**Supplementary Table 7.**

Gene deregulated with time between yMNs and mMNs in ^1^C9orf72 MNs (Patient 1) and ^2^C9orf72 (Patient 2) MNs versus control MNs (fold-change ≥ 1.5; adjusted P-Value ≤ 0.05). In ^1^C9orf72 versus control MNs, only one expressed gene named AJAP1 was downregulated. The AJAP1 protein was shown to participate in specific GABA receptor complexes [3] and interestingly GABA receptors were shown to be altered in the SOD1^G93A^ mouse model [4]. In ^2^C9orf72 versus control MNs, we identified one down-regulated gene MKNK1/MOBK3 coding for a protein that may have a role in the production of inflammatory mediators [5]. * This gene was kept as its fold-change was close to 1.5.

| **Gene**  **symbol** | **Gene Name** | **Fold Change** | **Adjusted**  **P-Value** |
| --- | --- | --- | --- |
| **Patient 1**  **AJAP1** | adherens junctions associated protein 1 | - 4.35 | 3.60x10^-2^ |
| **Patient 2**  **MKNK1//**  **MOB3C** | MAP kinase interacting serine/threonine kinase 1 // MOB kinase activator 3C | -1.47* | 4.84x10^-2^ |

**Supplementary Table 8.**

| **Gene symbol** | **Gene Name** | **Fold Change** | **Adjusted**  **P-Value** |
| --- | --- | --- | --- |
| **CRIM1** | cysteine rich transmembrane BMP regulator 1 | - 4.63 | 3.37x10^-14^ |
| **SNX21** | sorting nexin family member 21 | 1.77 | 5.76x10^-4^ |
| **PLCE1** | phospholipase C epsilon 1 | 5.03 | 1.02x10^-3^ |
| **GNB4** | G protein subunit beta 4 | 2.38 | 1.17x10^-3^ |
| **FAM3C** | family with sequence similarity 3 member C | 1.67 | 5.13x10^-3^ |
| **GPR83** | G protein-coupled receptor 83 | -3.84 | 6.39x10^-3^ |
| **NPTX1** | Neuronal pentraxin 1 | - 3.11 | 6.90x10^-3^ |
| **TEX2** | Testis expressed 2 | - 1.56 | 9.13x10^-3^ |
| **HLA-DMA** | Major histocompatibility complex, class II, DM alpha | - 2.71 | 1.01x10^-2^ |
| **PELI2** | pellino E3 ubiquitin protein ligase family member 2 | 2.02 | 1.30x10^-2^ |
| **CLIC5** | chloride intracellular channel 5 | 6.58 | 1.45x10^-2^ |
| **MAF** | MAF bZIP transcription factor | 3.13 | 1.98x10^-2^ |
| **BIK** | BCL2 interacting killer | 3.38 | 2.34x10^-2^ |
| **FZD7** | frizzled class receptor 7 | 3.54 | 3.24x10^-2^ |
| **ARHGAP22** | Rho GTPase activating protein 22 | 1.98 | 3.41x10^-2^ |
| **PLEKHB1** | pleckstrin homology domain containing B1 | 2.96 | 3.43x10^-2^ |
| **MOXD1** | monooxygenase DBH like 1 | 2.58 | 4.72x10^-2^ |

Gene deregulated with time between yMNs and mMNs in TARDBP MNs versus control MNs (fold-change ≥ 1.5; adjusted P-Value ≤ 0.05). Among these genes, PLEKHB1, encoding a protein with unknown cellular functions, was recently identified to be deregulated in MNs at motor symptom onset in TDP-43 driven ALS models [6].

**Supplementary Table 9: Excel file**

The excel file shows comparisons at each MN stage of differentiation (3 tabs showing results for pMNs, yMN and mMNs) of all ALS MNs versus control MNs. Analysis of these data and of deregulated genes are shown in Table 1.

1 Lee H, Ahn HH, Lee Wet al (2016) ENC1 Modulates the Aggregation and Neurotoxicity of Mutant Huntingtin Through p62 Under ER Stress. Mol Neurobiol 53: 6620-6634 Doi 10.1007/s12035-015-9557-8

2 Togawa J, Ohi T, Yuan JHet al (2019) Atypical Familial Amyotrophic Lateral Sclerosis with Slowly Progressing Lower Extremities-predominant Late-onset Muscular Weakness and Atrophy. Intern Med 58: 1851-1858 Doi 10.2169/internalmedicine.2222-18

3 Dinamarca MC, Raveh A, Schneider Aet al (2019) Complex formation of APP with GABAB receptors links axonal trafficking to amyloidogenic processing. Nat Commun 10: 1331 Doi 10.1038/s41467-019-09164-3

4 Carunchio I, Mollinari C, Pieri M, Merlo D, Zona C (2008) GAB(A) receptors present higher affinity and modified subunit composition in spinal motor neurons from a genetic model of amyotrophic lateral sclerosis. Eur J Neurosci 28: 1275-1285 Doi 10.1111/j.1460-9568.2008.06436.x

5 Buxade M, Parra-Palau JL, Proud CG (2008) The Mnks: MAP kinase-interacting kinases (MAP kinase signal-integrating kinases). Front Biosci 13: 5359-5373 Doi 10.2741/3086

6 Marques RF, Duncan KE (2022) SYNGR4 and PLEKHB1 deregulation in motor neurons of amyotrophic lateral sclerosis models: potential contributions to pathobiology. Neural Regen Res 17: 266-270 Doi 10.4103/1673-5374.317960
